# Adenosine Awakens Metabolism to Enhance Growth-Independent Killing of Tolerant and Persister Bacteria across Multiple Classes of Antibiotics

**DOI:** 10.1128/mbio.00480-22

**Published:** 2022-05-16

**Authors:** David A. Kitzenberg, J. Scott Lee, Krista B. Mills, Ju-Sim Kim, Lin Liu, Andrés Vázquez-Torres, Sean P. Colgan, Daniel J. Kao

**Affiliations:** a Molecular Biology Program, University of Colorado Anschutz Medical Campus, Aurora, Colorado, USA; b Mucosal Inflammation Program, University of Colorado Anschutz Medical Campus, Aurora, Colorado, USA; c Department of Medicine, University of Colorado Anschutz Medical Campus, Aurora, Colorado, USA; d Department of Immunology and Microbiology, University of Colorado Anschutz Medical Campus, Aurora, Colorado, USA; School of Medicine, Oregon Health & Science University

**Keywords:** *E. coli*, *Salmonella*, antibiotic resistance, nucleosides, persistence, tolerance

## Abstract

Metabolic and growth arrest are primary drivers of antibiotic tolerance and persistence in clinically diverse bacterial pathogens. We recently showed that adenosine (ADO) suppresses bacterial growth under nutrient-limiting conditions. In the current study, we show that despite the growth-suppressive effect of ADO, extracellular ADO enhances antibiotic killing in both Gram-negative and Gram-positive bacteria by up to 5 orders of magnitude. The ADO-potentiated antibiotic activity is dependent on purine salvage and is paralleled with a suppression of guanosine tetraphosphate synthesis and the massive accumulation of ATP and GTP. These changes in nucleoside phosphates coincide with transient increases in rRNA transcription and proton motive force. The potentiation of antibiotic killing by ADO is manifested against bacteria grown under both aerobic and anaerobic conditions, and it is exhibited even in the absence of alternative electron acceptors such as nitrate. ADO potentiates antibiotic killing by generating proton motive force and can occur independently of an ATP synthase. Bacteria treated with an uncoupler of oxidative phosphorylation and NADH dehydrogenase-deficient bacteria are refractory to the ADO-potentiated killing, suggesting that the metabolic awakening induced by this nucleoside is intrinsically dependent on an energized membrane. In conclusion, ADO represents a novel example of metabolite-driven but growth-independent means to reverse antibiotic tolerance. Our investigations identify the purine salvage pathway as a potential target for the development of therapeutics that may improve infection clearance while reducing the emergence of antibiotic resistance.

## INTRODUCTION

The immense clinical and economic burden of antimicrobial resistance (AMR) is projected to worsen. The rate at which AMR emerges outpaces the development of novel antibiotics, presenting a need for strategies to combat this growing global health threat (1–3). Antibiotic stewardship is a key component to targeting the development of AMR itself, but there is a need for novel therapeutic approaches as well. Subpopulations of antibiotic tolerant bacteria known as persisters play a critical role in the acquisition of AMR (4–6). Targeting antibiotic tolerant or persister bacteria could delay and combat the emergence of AMR (5). Stimulation with specific metabolites represents a fruitful strategy for the eradication of persisters (7).

Suppression of cellular energetics has been identified as one of the dominant mechanisms by which bacterial persistence develops in phylogenetically diverse bacteria (8–11). Disruption or decoupling of the proton motive force (PMF) from ATP synthesis increases the fraction of persistent bacteria in human pathogens (8–10). Perturbation of cellular energetics stimulates tolerance or persistence through multiple mechanisms. For example, inhibition of energetics decreases antibiotic uptake while suppressing replication, transcription, translation, cell division, and cell wall synthesis (8–13). Depletion of intracellular ATP also results in protein aggregation, promoting the less susceptible state of dormancy that has been shown to influence antibiotic tolerance (11). Conversely, chloramphenicol-treated, growth-arrested Salmonella demonstrates both increased ATP levels and antibiotic tolerance (14). It appears that under different conditions, metabolism or growth rate may provide a greater contribution toward antibiotic tolerance, and ultimately, the two may be uncoupled in certain conditions (15). Metabolite supplementation to potentiate antibiotic killing has been demonstrated against Escherichia coli and Staphylococcus aureus (7). Identification of specific metabolites that reverse growth-related antibiotic tolerance and persistence provides an opportunity to better understand the mechanisms of tolerance and persistence, which ultimately contribute to treatment-refractory infections and antibiotic resistance (6).

Adenosine (ADO) is involved in conserved and functionally diverse cellular processes. In the lumen of the human gut, ADO is generated in large quantities at the mucosal surface through the degradation of ATP by ectonucleotidases of epithelial cells (16). Enteric bacteria nearly universally transport extracellular ADO to the cytoplasm, where it is converted via phosphorolytic cleavage of the *N-*glycosyl bond to adenine or deaminated to inosine, which undergoes conversion to hypoxanthine and inositol monophosphate (17). We recently observed that exogenous ADO inhibits growth of nutritionally stressed Salmonella (16). Based on these observations, we tested whether ADO influences antibiotic killing of nutritionally stressed bacteria. Here, we report that salvage of extracellular ADO suppresses the stringent response, increases ATP and GTP levels, and promotes PMF and respiration, which together enhance killing of antibiotic tolerant and persister bacteria through growth-independent mechanisms.

## RESULTS

### ADO alters nucleotide metabolism during nutrient stress in E. coli.

**FIG 1:**
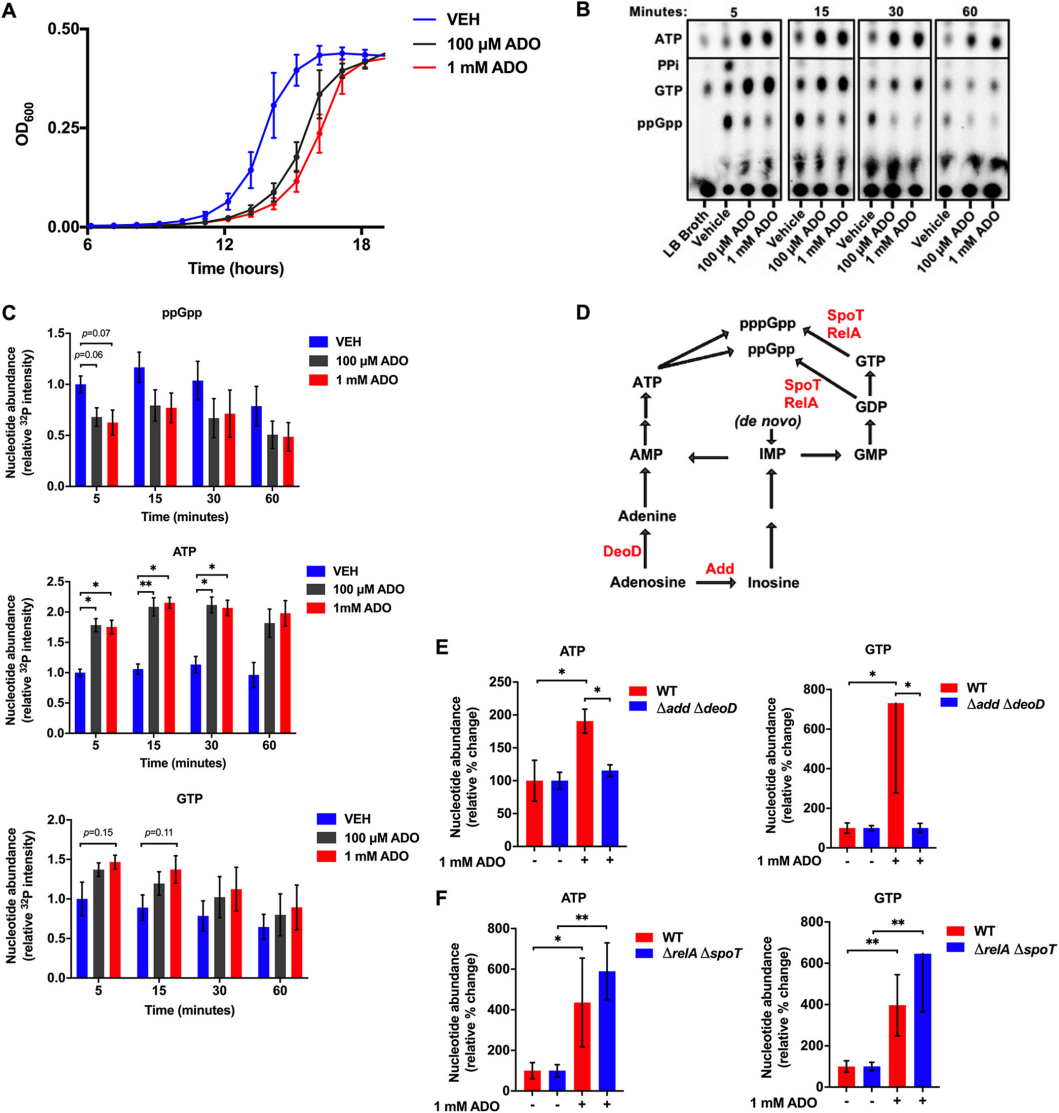
Extracellular ADO dysregulates nucleotide metabolism. (A) Growth of E. coli in M9 minimal medium supplemented with 0.4% glucose, treated at time zero with 100 μM or 1 mM ADO. (B) Representative thin-layer chromatography (TLC) autoradiogram of ^32^P-labeled nucleotide extracts from E. coli downshifted to M9 minimal medium supplemented with 0.4% glucose and treated at time zero with vehicle or ADO. Cells shifted to LB broth serve as a nutrient-rich control. (C) Relative changes in ATP, GTP, and ppGpp were determined by mean autoradiogram intensity of 3 replicate experiments from panel B. (D) Simplified purine salvage and (p)ppGpp synthesis pathways; enzymes of interest are shown in red. (E and F) HPLC analysis of intracellular nucleotide extracts 15 min after E. coli cells were downshifted from LB broth into PBS. Selected groups of cells were treated with ADO at time zero. Prior to treatment, experimental cultures were washed and diluted to equal starting CFU. Data are the mean of three biological replicates ± SEM. *, *P < *0.05; **, *P < *0.01, as assessed by two-way analysis of variance (ANOVA) with Tukey’s multiple-comparison and Mann-Whitney U tests. ADO, adenosine; VEH, vehicle control.

Exogenous ADO delays the growth of Salmonella (16) and E. coli (Fig. 1A) grown in glucose-containing M9 minimal media, suggesting that exogenous ADO has a significant influence on bacterial physiology. Casamino Acids rescue both Salmonella (16) and E. coli from the ADO-induced growth delay (Fig. S1A in the supplemental material), suggesting that ADO may interfere with the bacterial stringent response, a broadly conserved adaptive program that is required for bacterial growth during nutritional starvation (18–21). We measured the impact of ADO on the generation of the stringent response alarmones guanosine tetraphosphate and guanosine pentaphosphate [(p)ppGpp] in E. coli (18, 22). The stringent response was induced through downshift of ^32^P-labeled E. coli grown in amino acid-supplemented morpholinepropanesulfonic acid (MOPS) minimal medium into amino acid-free M9 minimal medium. Guanosine tetraphosphate accumulated after 5 min of the downshift. In contrast, E. coli shifted from ^32^P-containing amino acid-supplemented MOPS minimal media into nutrient-rich LB broth did not accumulate ppGpp (Fig. 1B). During amino acid starvation, ppGpp accumulates more than pppGpp (23). The downshift did not trigger the accumulation of pppGpp, as has been published in response to other stressors (24). Addition of 100 μM or 1 mM ADO attenuated ppGpp accumulation for at least 60 min after downshift. Though (p)ppGpp is hydrolyzed to GDP/GTP and PP_i_, we did not observe an increase in PP_i_ in the ADO-treated groups (Fig. 1B), suggesting that ADO does not reduce ppGpp accumulation through enhanced hydrolysis.

10.1128/mbio.00480-22.1 FIG S1 Impact of ADO on growth. (A) Growth of E. coli in M9 minimal medium treated at time zero with ADO and 0.2% Casamino Acids. (B) Growth of wild-type and Δ*add* Δ*deoD* mutant E. coli in M9 minimal medium supplemented with 0.4% glucose, treated at time zero with 1 mM ADO. Data are the mean of three biological replicates ± SEM. *, *P < *0.05, as assessed by two-way ANOVA with Tukey’s multiple comparison. ADO, adenosine; VEH, vehicle control. Download FIG S1, TIF file, 0.2 MB. Copyright © 2022 Kitzenberg et al. 2022 Kitzenberg et al. https://creativecommons.org/licenses/by/4.0/ This content is distributed under the terms of the Creative Commons Attribution 4.0 International license.

The stringent response activates transcription of amino acid biosynthesis genes (18). We hypothesized that inhibition of the stringent response would result in dysregulation of amino acid biosynthesis. To investigate this, we measured concentrations of free amino acids in bacterial lysates after downshift into amino acid-free media (25). The addition of ADO resulted in significant decreases in the intracellular concentrations of arginine, isoleucine, and phenylalanine. Aspartate, glutamate, histidine, leucine, lysine, and methionine also decreased; however, these decreases did not achieve statistical significance (*P < *0.05) (Table S1). Together with our observation that amino acid supplementation complements the ADO-induced growth defect, our data suggest that ADO treatment promotes amino acid dysregulation and depletes select amino acids, which may delay bacterial growth (23).

10.1128/mbio.00480-22.8 TABLE S1 Intracellular amino acid quantification by LC-MS. Bacteria were subcultured 1:50 from an overnight stock. Subcultures were grown for ~2.5 h until late exponential phase. One milliliter of bacteria was then washed three times in PBS and resuspended into PBS to an OD_600_ of 0.4. Cells were treated for 15 minutes at 37°C, 250 rpm. Following treatment, bacteria were pelleted, supernatant was aspirated, and cell pellets were extracted at 8 × 10^9^ cells per mL of lysis/extraction buffer (methanol/acetonitrile/water, 5:3:2 [vol/vol], containing 1 μM of a commercial amino acid standard mix [Cambridge Isotope Laboratories]). Extracts were analyzed as previously described (23). Data are the mean of three biological replicates. *, *P < *0.05, as assessed by Student’s *t* test. Download Table S1, PDF file, 0.04 MB. Copyright © 2022 Kitzenberg et al. 2022 Kitzenberg et al. https://creativecommons.org/licenses/by/4.0/ This content is distributed under the terms of the Creative Commons Attribution 4.0 International license.

In addition to suppressing ppGpp accumulation, ^32^P autoradiography revealed that the addition of ADO resulted in concomitant and sustained increases in ATP and GTP (Fig. 1B and C). The observed ATP and GTP accumulation raised the possibility that the phenotypes noted after the addition of ADO may involve purine salvage. After uptake, ADO is first metabolized to inosine or adenine by adenosine deaminase (Add) or purine nucleoside phosphorylase (DeoD), respectively (Fig. 1D) (17). To disrupt ADO salvage, we generated an E. coli strain deficient in *add* and *deoD* (Δ*add* Δ*deoD*). Unlike the wild-type strain, this purine salvage-deficient mutant was immune to the growth delay effects of ADO in glucose-containing M9 minimal media (Fig. S1B). Neither ATP nor GTP accumulated in this salvage-deficient strain when exposed to ADO, demonstrating that purine salvage is necessary for ADO-elicited increases in ATP and GTP (Fig. 1E). Unlike the salvage-deficient mutant, a (p)ppGpp-null Δ*relA* Δ*spoT* strain experienced significant increases in ATP and GTP following treatment with ADO (Fig. 1F). Thus, extracellular ADO fundamentally impacts nucleotide metabolism.

### ADO antagonizes markers of persister bacteria.

Physiologic markers important for bacterial tolerance and persistence have been previously described (10). Shan et al. showed that several amino acid auxotrophs were less tolerant to gentamicin because of increases in the PMF (26). Accumulation of (p)ppGpp and depletion of ATP have both been associated with persister formation (8, 27). Because ADO suppresses the stringent response while concurrently increasing intracellular ATP and GTP, we suspected that ADO might influence phenotypes that are characteristic of persister bacteria. To test this, we examined multiple markers associated with persister bacteria.

**FIG 2:**
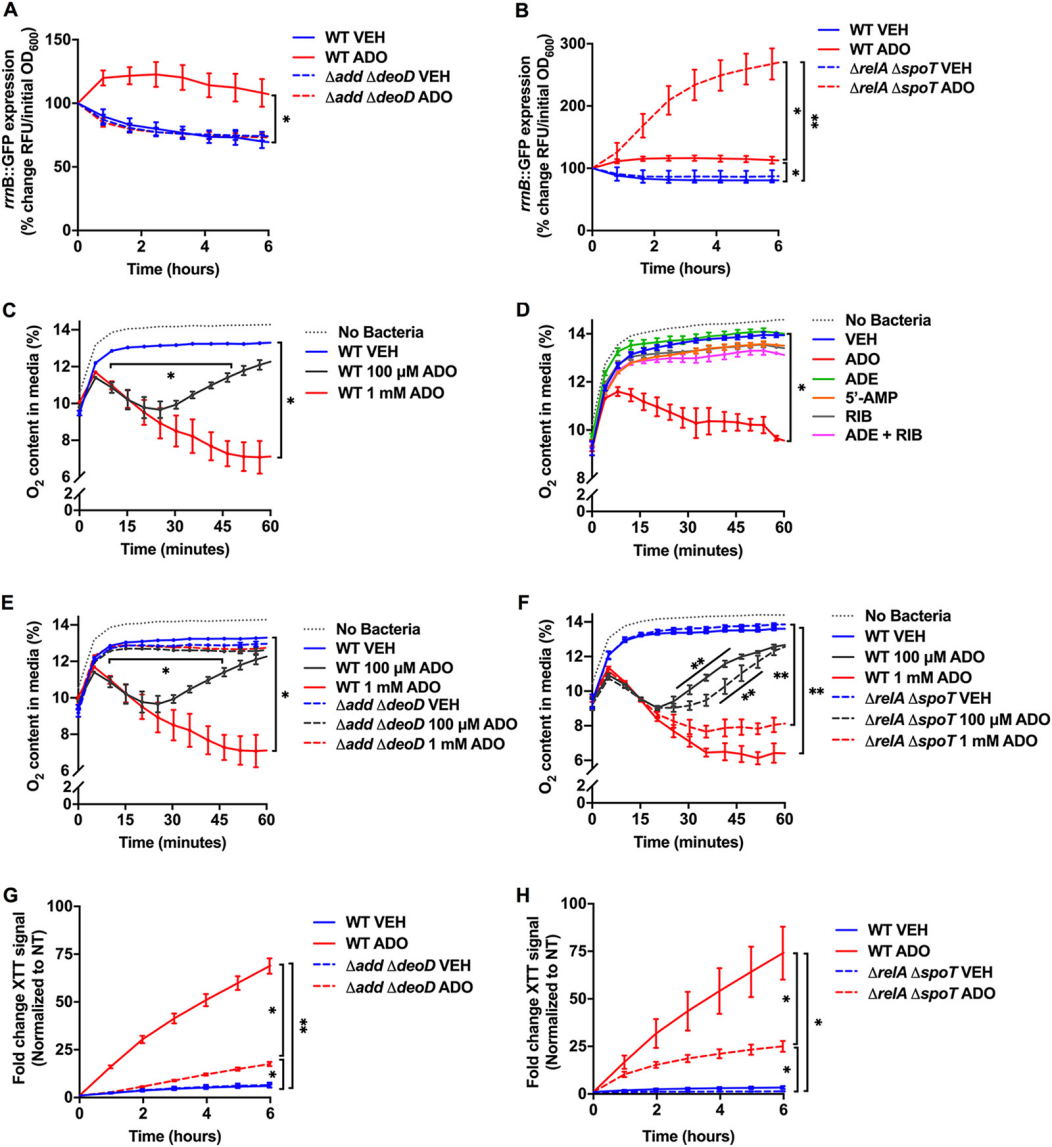
ADO hyperstimulates rRNA transcription, O_2_ respiration, and electron transport chain activity. (A and B) *rrnB* P1:GFP signal from wild-type and mutant E. coli following the downshift from LB broth to PBS. Selected samples were treated with 1 mM ADO at time zero. Fluorescent signal and OD_600_ were monitored over time at 37°C. Signal was normalized to initial inoculum. (C to F) O_2_ consumption was examined in PBS with E. coli at 37°C in a shaker incubator using PreSens OxoDish. Dotted gray line indicates O_2_ content in cell-free media. (G and H) Tetrazolium dye, XTT, in PBS; for each time point, the signal was normalized to the time zero of the wild-type vehicle control group. Readings were taken every 10 min at 37°C. No growth was observed under experimental conditions in panels A to H. Data are the mean of three biological replicates ± SEM. *P, < 0.05; **, *P < *0.01, as estimated by two-way ANOVA with Tukey’s multiple comparison. ADE, adenine; ADO, adenosine; RIB, d-ribose; RFU, relative fluorescent unit; VEH, vehicle control.

The expression of 16S rRNA promoter *rrnB* P1, which is negatively regulated by (p)ppGpp and positively regulated by ATP, has been inversely correlated with both antibiotic tolerance and bacterial persistence (8, 28–30). To investigate the influence of ADO on *rrnB* P1 promoter activity, an E. coli strain harboring an *rrnB:gfp* reporter plasmid was grown in LB broth to mid-log phase and then downshifted into phosphate-buffered saline (PBS). Instead of M9, we used PBS to maintain equal numbers of bacteria over the extent of the experiment, as ADO can cause growth delay in M9 (Fig. 1A) (16). Upon downshift of the reporter strain into PBS, there was a rapid decrease in promoter activity (Fig. 2A), consistent with either a rise in (p)ppGpp or a decrease in ATP (Fig. 1B). In contrast, downshift in the presence of 1 mM ADO resulted in a rapid increase in promoter activity (Fig. 2A). To determine whether the increase in reporter activity was due to changes in ATP or (p)ppGpp, we used Δ*add* Δ*deoD rrnB:gfp* and Δ*relA* Δ*spoT rrnB:gfp* reporter strains. ADO did not induce *rrnB:gfp* expression in the Δ*add* Δ*deoD* reporter strain (Fig. 2A), suggesting that *rrnB* P1 promoter activity is dependent upon synthesis of ATP through the purine salvage pathway. In sharp contrast, downshift of the (p)ppGpp-null reporter strain in the presence of ADO resulted in a large increase in *rrnB* P1 promoter activity (Fig. 2B). These findings are consistent with known antagonistic roles of ATP and (p)ppGpp in ribosomal promoter activity: whereas ATP is the initiating nucleotide at six of the seven *rrn* P1 promoters (including *rrnB*), ppGpp shortens the half-life of open complexes at the *rrn* P1 promoter (30). We also examined the influence of ADO on transcription of the *rrnD* P1 promoter, whose activity is increased by GTP (30). As expected, ADO stimulated *rrnD* P1 promoter activity, although the changes in activity were smaller in magnitude than the *rrnB* P1 promoter (Fig. S2A), possibly due to inherent differences in promoter strength or differences in absolute nucleotide concentrations (Fig. S2B). Together, these observations suggest that the ADO-induced increase in ribosomal promoter activity is primarily due to the observed increases in ATP and GTP.

10.1128/mbio.00480-22.2 FIG S2 ADO-driven changes in rRNA transcription and nucleotide concentration in mutant E. coli. (A) *rrnD* P1:GFP signal from control and mutant E. coli following PBS downshift and treatment with 1 mM ADO at time zero. Fluorescent signal and OD_600_ were monitored kinetically at 37°C with agitation. The Δ*relA* Δ*spoT* strain consistently showed more variability between experiments relative to other strains tested. (B) HPLC analysis of E. coli intracellular nucleotide extracts 15 minutes after medium washout and downshift into PBS with vehicle or 1 mM ADO treatment at time zero. Values based on area under the curve. For curve analysis, the analyzer was blinded to sample IDs. Data are the mean of three biological replicates ± SEM. *, *P < *0.05, as assessed by two-way ANOVA with Tukey’s multiple comparison and one-way ANOVA followed by Fisher’s LSD test for panels A and B, respectively. ADO, adenosine; RFU, relative fluorescent unit; VEH, vehicle control. Download FIG S2, TIF file, 0.4 MB. Copyright © 2022 Kitzenberg et al. 2022 Kitzenberg et al. https://creativecommons.org/licenses/by/4.0/ This content is distributed under the terms of the Creative Commons Attribution 4.0 International license.

Antibiotic tolerance of persister cells has been linked to decreased cellular respiration (9). To determine whether ADO influences aerobic cellular respiration in E. coli, we measured O_2_ consumption using PreSens OxoDish (31). Baseline dissolved O_2_ concentrations in the media were low because measurements were taken at approximately 5,391 feet above sea level. Bacteria downshifted from LB broth to PBS did not deplete O_2_ from the media (Fig. 2C). In stark contrast, ADO-treated E. coli depleted O_2_ in a dose-dependent manner (Fig. 2C). We then examined the effect that upstream and downstream ADO metabolites have on respiration. Strikingly, 1 mM adenine, 5′-AMP, d-ribose, or a combination of adenine and d-ribose did not stimulate aerobic respiration (Fig. 2D).

We then assessed O_2_ consumption in the purine salvage and (p)ppGpp-null mutants. Unlike the wild-type strains, the Δ*add* Δ*deoD* mutant did not deplete O_2_ in the presence of 100 μM or 1 mM ADO, suggesting that purine salvage is necessary for promotion of cellular respiration by ADO (Fig. 2E). In contrast, when the (p)ppGpp-null mutant was treated with ADO, there was a dose-dependent increase in respiration, similar to that seen in the wild-type strain (Fig. 2F).

To see if ADO-induced O_2_ consumption correlates with increased PMF and oxidative phosphorylation, we examined electron transport chain (ETC) activity using the tetrazolium salt XTT {2,3-bis(2-methoxy-4-nitro-5-sulfophenyl)-5-((phenylamino)carbonyl)-2H-tetrazolium hydroxide} (32). Reduction of XTT to the colorimetric formazan product occurs in the setting of increased ETC activity (33). Bacteria were downshifted from LB broth to PBS and treated with vehicle or 1 mM ADO in the presence of XTT. The downshift revealed a low basal level of XTT conversion in untreated E. coli, supporting our previous finding that minimal O_2_ consumption occurs in starvation conditions (Fig. 2G). When controls were downshifted in the presence of 1 mM ADO, a significant >50-fold increase in XTT conversion was noted (Fig. 2G) (*P* < 0.01). The magnitude of XTT conversion was significantly lower in the Δ*add* Δ*deoD* mutant than the wild-type control (Fig. 2G) (*P* < 0.05). A Δ*relA* Δ*spoT* E. coli strain sustained significant increases in XTT conversion, although to a lower extent than wild-type controls (Fig. 2H). The increased XTT reduction seen after ADO suggests stimulation of aerobic respiration.

In summary, exogenous ADO treatment of nutritionally starved E. coli decreases ppGpp accumulation and increases ATP and GTP. These changes in nucleotide metabolism correlate with antagonism of multiple markers of persister cells, including increased rRNA transcription, increased ETC activity, and increased O_2_ consumption. Based on this experimental evidence and the role that PMF and energetics play in antibiotic killing (8–10, 12, 34), we tested whether ADO treatment influences antibiotic lethality.

### ADO potentiates the bactericidal activity of different classes of antibiotics against E. coli.

**FIG 3:**
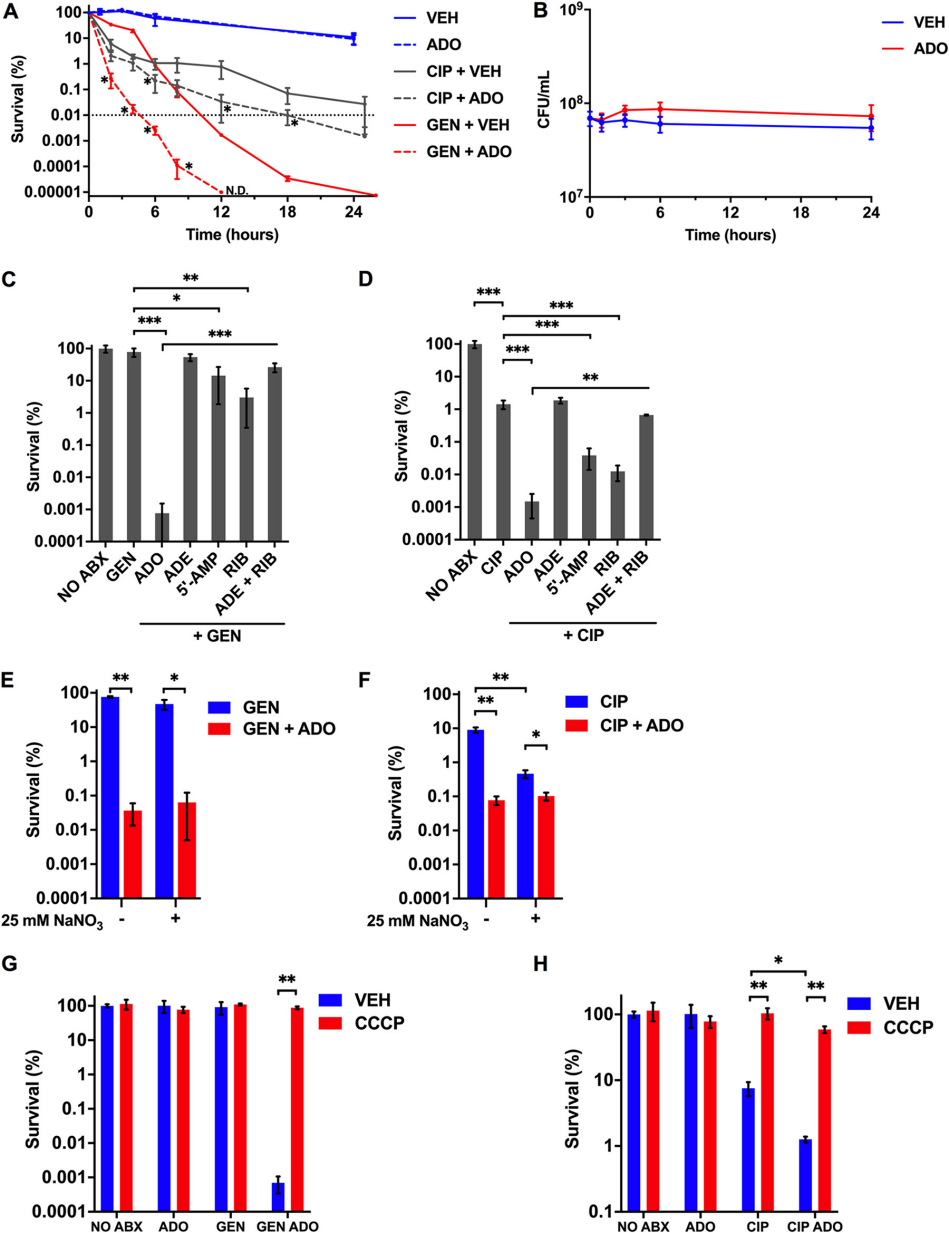
ADO potentiates antibiotic lethality. (A) E. coli grown to stationary phase in LB broth was treated with very high concentrations of antibiotics for 0 to 24 h. Selected groups were treated with 80 μg/mL GEN, 3 μg/mL CIP, and/or 1 mM ADO. (B) E. coli downshifted from LB broth into PBS and treated at time zero with 1 mM ADO. Both control and treatment groups were established from one culture to ensure equal starting CFU for each group within an experiment. (C and D) Survival of E. coli after downshift from exponential-phase LB broth to PBS followed by an 18 h treatment with 5 μg/mL GEN or 500 ng/mL CIP. Selected samples were treated with 1 mM of the indicated nucleotides or metabolites. (E and F) Eighteen-hour survival experiment performed in an anaerobic chamber. Bacteria were passaged for at least 1 week to adapt to anaerobic conditions. Prior to treatment, bacteria were shifted from LB broth to PBS. Sodium nitrate (25 mM) was added at time of antibiotic treatment. Selected samples were treated with 5 μg/mL GEN, 500 ng/mL CIP, and/or 1 mM ADO. (G and H) Survival of aerobic E. coli after downshift from exponential-phase LB broth to PBS followed by a 3 h treatment with 5 μg/mL GEN or 500 ng/mL CIP. Selected samples were treated with 1 mM ADO and 50 μM CCCP at time of antibiotic treatment. Data are the mean of three biological replicates ± SEM. Dotted line indicates MDK_99.99_ threshold. *, *P < *0.05; **, *P < *0.01; ***, *P < *0.001, as assessed by Student's *t* test, Mann-Whitney U test, or one-way ANOVA with Fisher’s least significant difference (LSD) multiple comparison at each time point. ADE, adenine; ADO, adenosine; RIB, d-ribose; VEH, vehicle control.

Following our observations that ADO counters markers of persister cells such as respiration and ETC activity, we examined the effects of ADO on the susceptibility of persister cells to antibiotics. Persister cells form stochastically, can be induced in response to environmental stimuli (35), and are also found in relatively high numbers during the stationary phase (3). Thus, to characterize the influence of ADO under conditions enriched for persisters, we determined the survival of stationary-phase E. coli cultures after addition of antibiotics at ≥20 times the MIC (Table S2A) (3). The minimum duration for killing (MDK) curve is the preferred metric for quantifying antibiotic tolerant and persister bacteria in a population, with a biphasic kill curve indicating the presence of persisters (36). The minimum duration for killing 99.99% of the population (MDK_99.99_) curves demonstrated that the addition of 1 mM ADO together with 80 μg/mL gentamicin (GEN) or 3 μg/mL ciprofloxacin (CIP) resulted in significantly (*P < *0.05) more killing of stationary-phase E. coli than antibiotics alone (Fig. 3A). GEN-treated bacteria experienced a relatively constant kill rate from 0 to 18 h and a slower kill rate from 18 to 24 h. The slower killing phase may represent the antibiotic activity against a persister subpopulation (36). The addition of ADO caused a dramatic leftward shift in the GEN kill curve (Fig. 3A). Furthermore, ADO treatment suppressed a second lower rate of killing in the GEN kill curve, suggesting this nucleoside promotes killing of the persister population. The CIP MDK_99.99_ curves revealed markedly different killing kinetics from GEN. CIP and CIP-ADO groups experienced similar killing rates in the population during the first 4 h (Fig. 3A). The kill curves then shallow out, indicative of biphasic killing. The MDK_99.99_ was not reached in the CIP-treated group, but with ADO treatment, the MDK_99.99_ shifted left to 18 h, suggesting enhanced killing of persisters (Fig. 3A). Importantly, ADO alone did not alter growth or survival of the stationary cultures, so the major effect of ADO on antibiotic killing was likely not due to stimulation of growth by ADO or inherent antimicrobial activity of ADO. Together, these results indicate that ADO potentiates GEN- and CIP-mediated killing of persister cells.

10.1128/mbio.00480-22.9 TABLE S2 Strain information. (A) Minimum inhibitory concentrations. (B) Strains. (C) Plasmids. Download Table S2, PDF file, 0.04 MB. Copyright © 2022 Kitzenberg et al. 2022 Kitzenberg et al. https://creativecommons.org/licenses/by/4.0/ This content is distributed under the terms of the Creative Commons Attribution 4.0 International license.

Having shown that ADO enhances antibiotic-mediated killing under conditions enriched for persister cells, we turned to conditions that would promote antibiotic tolerance. Specifically, E. coli cells grown to exponential phase in LB broth were downshifted to PBS to induce a growth-restricted starvation state. Accumulation of ppGpp inhibits cell growth; however, E. coli can continue to divide for 90 min after induction of ppGpp (37). Growth can be an important determinant of tolerance and antibiotic killing. Therefore, we assessed the impact of ADO on growth under the PBS-downshifted conditions used in our investigations. ADO did not significantly increase growth or compromise survival compared to untreated controls over a 24-h period (Fig. 3B). To further assess the effects ADO has on growth and killing, we assessed survival of E. coli after 18 h of treatment with 40 μg/mL ampicillin because β-lactam killing is proportional to growth rate (38). The combination of ADO with ampicillin slightly improved survival compared to ampicillin alone, suggesting that the primary mechanism of ADO-driven antibiotic potentiation is unrelated to growth stimulation (Fig. S3).

10.1128/mbio.00480-22.3 FIG S3 ADO fails to potentiate β-lactam antibiotics. Survival of E. coli after downshift from exponential-phase LB broth to PBS followed by an 18 h treatment with 40 μg/mL ampicillin. Selected samples were treated with 1 mM ADO. Data are the mean of four biological replicates ± SEM. *, *P < *0.05, as assessed by Student’s *t* test. ADO, adenosine; VEH, vehicle control. Download FIG S3, TIF file, 0.05 MB. Copyright © 2022 Kitzenberg et al. 2022 Kitzenberg et al. https://creativecommons.org/licenses/by/4.0/ This content is distributed under the terms of the Creative Commons Attribution 4.0 International license.

In the presence of 1 mM ADO, treatment with 5 μg/mL GEN or 500 ng/mL CIP resulted in a significant (*P < *0.001) reduction in survival compared to controls treated with only antibiotics. ADO enhanced GEN and CIP killing of E. coli by 10^5^-fold and 10^3^-fold, respectively (Fig. 3C and D). Both 5′-AMP and d-ribose enhanced killing by GEN and CIP (*P < *0.05); however, the magnitude was substantially smaller than ADO. The combination of adenine and d-ribose did not further decrease survival (Fig. 3C and D). Yang et al. investigated the enhancement of antibiotic lethality against E. coli using purines and pyrimidines, including adenine but not ADO (39). While adenine reduces O_2_ consumption and fails to potentiate antibiotic lethality (39), our investigations here show that ADO greatly enhances both aerobic respiration and lethality of GEN and CIP. The differences between adenine and ADO might be related to the PMF and respiration-enhancing activity of the latter.

Bacteria encounter a gradient of O_2_ concentrations within tissue microenvironments (40, 41). To assess whether the influence of ADO on GEN- and CIP-mediated killing is an O_2_-dependent process, we performed the killing assays under anaerobic conditions in the presence or absence of the alternative terminal electron acceptor nitrate (NO_3_^−^). ADO treatment alone did not influence survival in anaerobic conditions (Fig. S4A). GEN reduced survival of anaerobic E. coli to 75%, while the addition of NO_3_^−^ reduced survival to 47%. In sharp contrast, ADO enhanced GEN killing roughly 1,000-fold regardless of the presence of NO_3_^−^ (Fig. 3E). The addition of NO_3_^−^ to CIP significantly (*P < *0.05) reduced survival of anaerobic E. coli from 9% to 0.5%. The addition of ADO to CIP significantly reduced survival to 0.08% and 0.1% in the absence and presence of NO_3_^−^, respectively (Fig. 3F). To further characterize the impact of ADO under anaerobic conditions, we examined XTT conversion and intracellular ATP and succinate concentrations by liquid chromatography-mass spectrometry (LC-MS). Treatment of E. coli with 1 mM ADO in the absence or presence of NO_3_^−^ significantly increased XTT conversion over a 2-h period (Fig. S4B). Following a 15-min treatment with 1 mM ADO, ATP levels increased but did not reach statistical significance in the absence of NO_3_^−^ (*P = *0.072) (Fig. S4C). However, in the presence of NO_3_^−^, ATP levels significantly increased (*P < *0.05) (Fig. S4C). We observed a significant (*P < *0.05) increase in succinate concentrations in the absence but not in the presence of NO_3_^−^, suggesting that fumarate may serve as an alternative electron acceptor when NO_3_^−^ is absent (Fig. S4C). Together, these results indicate that reactive oxygen and nitrogen species are not primary mechanisms by which ADO enhances antibiotic killing. In addition, these findings indicate that under anaerobic conditions, ADO increases ETC activity and can potentiate GEN and CIP killing independently of O_2_ or NO_3_^−^ respiration.

10.1128/mbio.00480-22.4 FIG S4 Impact of ADO on survival, ETC activity, ATP, and succinate under anaerobic conditions. For all experiments, E. coli was repeatedly passaged in anaerobic chamber to adapt to anaerobic conditions. (A) Eighteen-hour survival experiment performed in an anaerobic chamber. Prior to treatment, bacteria were shifted from LB broth to PBS. Sodium nitrate (25 mM) was added at the time of ADO treatment. Selected samples were treated with 1 mM ADO and sodium nitrate. (B) Two-hour XTT experiment performed in an anaerobic chamber. Prior to treatment, bacteria were shifted from LB broth to PBS containing XTT. Bacteria were treated with vehicle control or 1 mM ADO. For select samples, sodium nitrate (25 mM) was added at time of treatment. (C) Anaerobic LC-MS analysis of intracellular ATP and succinate. Cells were downshifted from LB broth into PBS. E. coli was treated with vehicle control or 1 mM ADO, and 15 minutes after treatment, samples were snap frozen and extracted. For select samples, sodium nitrate (25 mM) was added at the time of treatment. Data are the mean of three biological replicates ± SEM. *, *P < *0.05; **, *P < *0.01, as assessed by Student’s *t* test (panels A and B) and one-way ANOVA followed by Fisher’s LSD test (panel C). ADO, adenosine; NO_3_^−^, sodium nitrate; VEH, vehicle control. Download FIG S4, TIF file, 0.4 MB. Copyright © 2022 Kitzenberg et al. 2022 Kitzenberg et al. https://creativecommons.org/licenses/by/4.0/ This content is distributed under the terms of the Creative Commons Attribution 4.0 International license.

Next, we tested whether ADO promotes increased killing through increased PMF and cellular respiration. We utilized the protonophore carbonyl cyanide *m*-chlorophenyl hydrazone (CCCP) to uncouple oxidative phosphorylation and abolish PMF. The addition of CCCP abolished the lethality-enhancing effect of ADO for GEN and CIP (Fig. 3G and H). We also found that ADO increases uptake of GEN (Fig. S5). These results, along with XTT observations, support that ADO enhances antibiotic lethality by enhancing ETC activity and PMF.

10.1128/mbio.00480-22.5 FIG S5 Gentamicin uptake after ADO treatment. Bacteria were subcultured 1:50 from an overnight stock. Subcultures were grown for ~3 h until late-exponential phase. Bacteria were then washed twice in PBS and then diluted to an OD_600_ of 0.25 in PBS. Treatment groups were divided, and [^3^H]gentamicin was added to a final concentration of 10 μCi. Bacteria were incubated at 37°C with agitation for 30 minutes. Selected samples were treated with 1 mM ADO. Samples were then added to a vacuum filter and washed to remove extracellular [^3^H]gentamicin. Filters were then placed in scintillation vials, and a 1-min continuous [^3^H]cpm scintillation count was taken. Data are the means of three biological replicates. *P < 0.05 as assessed by Student’s t-test. ADO, adenosine; VEH, vehicle control. Download FIG S5, TIF file, 0.05 MB. Copyright © 2022 Kitzenberg et al. 2022 Kitzenberg et al. https://creativecommons.org/licenses/by/4.0/ This content is distributed under the terms of the Creative Commons Attribution 4.0 International license.

### The minimum duration for killing in E. coli increases with the loss of purine salvage or the stringent response.

**FIG 4:**
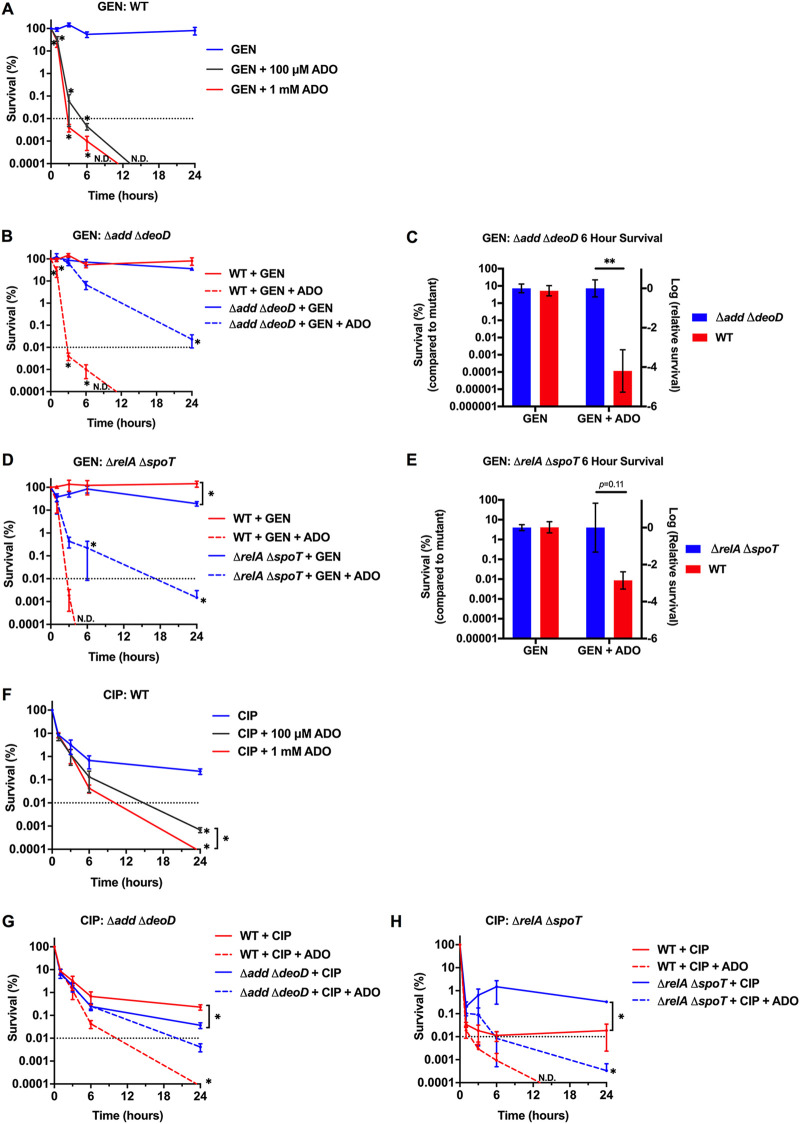
ADO shortens MDK_99.99_ duration. (A, B, D, and F to H) Survival of E. coli after downshift from exponential-phase LB broth to PBS followed by 0 to 24 h treatment to establish MDK values. Samples were treated with 5 μg/mL GEN or 500 ng/mL CIP. (C and E) Figures are data from MDK curves presented in panels B and D, respectively. Control strain survival values after 6 h of treatment were normalized to the mutant strain. For all experiments, ADO did not cause significant changes in growth or survival compared to VEH. Data are the mean of three biological replicates ± SEM. *, *P < *0.05; **, *P < *0.01; ***, *P < *0.001, as assessed by one-way ANOVA with Fisher’s LSD multiple comparison at each time point or Student's *t* test. Dotted line indicates MDK_99.99_ threshold. Asterisk denotes significant difference between treatment groups within a strain unless indicated by a bracket for between-strain comparisons. ADO, adenosine; ND, no CFU detected; VEH, vehicle control.

To establish MDK_99.99_ curves, we examined E. coli survival over time with antibiotic treatments in growth-restricted PBS conditions. We observed 80% survival of E. coli after exposure to 5 μg/mL GEN for 24 h; no viable bacteria were detectable at 24 h when 100 μM or 1 mM ADO was included with GEN (Fig. 4A). Coadministration of ADO with GEN resulted in a dramatic shift of the MDK_99.99_ for both wild-type control strains (Fig. 4B and D). The dose-dependent shift in the kinetics of bacterial killing for GEN suggests that exogenous ADO reduces starvation-induced antibiotic tolerance.

To investigate the role of purine salvage and the stringent response on ADO-potentiated GEN killing, we measured survival of an Δ*add* Δ*deoD* mutant after GEN treatment. ADO was less effective at potentiating GEN in the Δ*add* Δ*deoD* mutant than the wild type (Fig. 4B and C). Moreover, the MDK_99.99_ for GEN and ADO was approximately 3 h in the wild-type control strain but did not achieve 99.99% killing of the mutant strain after 24 h under the same conditions (Fig. 4B). These dramatic shifts in MDK_99.99_ suggest that purine salvage is a major determinant of the influence of ADO on GEN lethality. Significant ADO-potentiated GEN killing was observed in the Δ*add* Δ*deoD* mutant at 24 h (Fig. 4B). The delayed killing kinetics suggest that ADO may be salvaged less efficiently through redundant purine salvage enzymes. The *rihC* gene encodes a low expressed, constitutive ribonucleoside hydrolase that converts ADO to adenine (17, 42). This alternative pathway may be responsible for the intermediate ADO phenotype seen in the purine salvage mutant at 24 h. To verify that the observed loss of phenotype in the purine salvage mutant was specific to the disruption of *add* or *deoD*, we complemented the mutant with the low-copy-number pWSK29 vector expressing the *add* or *deoD* genes under the control of their native promoters. Based on the MDK killing kinetics (Fig. 4B and C), the mutants were challenged with 5 μg/mL GEN in the presence or absence of 1 mM ADO for 6 h in PBS. The Δ*add* Δ*deoD* pWSK29 empty vector and the Δ*add* Δ*deoD* pWSK29::*add* mutants demonstrated comparable survival to the Δ*add* Δ*deoD* mutant. However, the expression of *deoD* restored the susceptibility of Δ*add* Δ*deoD* E. coli to the effects of GEN and ADO to levels recorded in the wild-type strain (Fig. S6). Conversion of ADO to inosine through *add* is the predominant initial step in ADO salvage (42). The pathway then relies on DeoD to convert inosine to hypoxanthine and ADO to adenine (17). Our investigations indicate that the conversion of these nucleosides to the corresponding nucleobases is critical for the potentiation of GEN killing by ADO.

10.1128/mbio.00480-22.6 FIG S6 Survival differences in complemented purine salvage mutants. Survival of wild type, Δ*add* Δ*deoD* mutant, and pWSK29::*add*- or pWSK29::*deoD*-complemented Δ*add* Δ*deoD* mutant E. coli after downshift from exponential-phase LB broth to PBS followed by a 6-h treatment with 5 μg/mL GEN. Selected samples were treated with 1 mM ADO. Data are the mean of three biological replicates ± SEM *, *P < *0.05; **, *P < *0.01, as assessed by one-way ANOVA with Fisher’s LSD multiple comparison. ADO, adenosine; EV, empty vector; VEH, vehicle control. Download FIG S6, TIF file, 0.1 MB. Copyright © 2022 Kitzenberg et al. 2022 Kitzenberg et al. https://creativecommons.org/licenses/by/4.0/ This content is distributed under the terms of the Creative Commons Attribution 4.0 International license.

When the Δ*relA* Δ*spoT* mutant was treated with GEN and ADO, the MDK_99.99_ was achieved at 17 h compared to 3 h for the wild-type control strain (Fig. 4D). The MDK_99.99_ findings highlight an important role for the stringent response in ADO-driven enhanced lethality of GEN against growth-arrested tolerant bacteria. However, it appears that the ability of ADO to potentiate GEN may be more dependent on purine salvage than the stringent response (Fig. 4C and E).

Building on our observation that ADO enhances CIP killing at 18 h, we established CIP MDK_99.99_ curves. Addition of 100 μM or 1 mM ADO to 500 ng/mL CIP resulted in significant (*P < *0.05) killing after 24 h compared to CIP-treated controls (Fig. 4F). The Δ*add* Δ*deoD* mutant had improved survival when treated with CIP and ADO compared to the control strain (Fig. 4G). These observations indicate that ADO enhanced lethality is partially, but not fully, dependent on the purine salvage pathway. The Δ*relA* Δ*spoT* mutant was less susceptible to CIP than its isogenic E. coli, which may be due to experimental conditions. The MDK_99.99_ of the Δ*relA* Δ*spoT* mutant treated with CIP and ADO was 6 h compared to 2 h in the wild-type control strain (Fig. 4H). The prolonged MDK_99.99_ observed in the Δ*relA* Δ*spoT* mutant suggests that ADO-driven disruption of stringent response contributes to enhanced CIP lethality.

### ADO potentiates antibiotic lethality against Salmonella enterica serovar Typhimurium.

To examine whether the impact of ADO on antibiotic lethality could be applied to other Gram-negative bacteria, we subjected Salmonella enterica serovar Typhimurium strain 14028s to GEN or CIP in the presence of ADO. ADO treatment had similar enhancement of GEN and CIP MDK_99.99_ values (Fig. S7).

10.1128/mbio.00480-22.7 FIG S7 *S.* Typhimurium survival following ADO and antibiotic treatment. Survival of *S.* Typhimurium after downshift from exponential-phase LB broth to PBS followed by 0 to 24 h treatment with antibiotics to establish MDK values. Samples were treated with 5 μg/mL GEN or 500 ng/mL CIP. Data are the mean of three biological replicates ± SEM. *, *P < *0.05, as assessed by one-way ANOVA with Fisher’s LSD multiple comparison at each time point. Dotted line indicates MDK_99.99_ threshold. Asterisk denotes significant difference between treatment group and control within a strain unless indicated by a bracket for 100 μM versus 1 mM ADO treatment comparison. ADO, adenosine; ND, no CFUs detected; VEH, vehicle control. Download FIG S7, TIF file, 0.2 MB. Copyright © 2022 Kitzenberg et al. 2022 Kitzenberg et al. https://creativecommons.org/licenses/by/4.0/ This content is distributed under the terms of the Creative Commons Attribution 4.0 International license.

**FIG 5:**
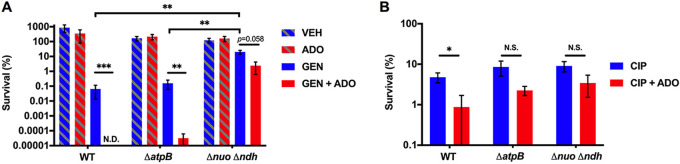
ADO promotes antibiotic killing of *S.* Typhimurium. (A and B) Eighteen-hour survival of *S.* Typhimurium grown to stationary phase in LB broth followed by treatment with 50 μg/mL GEN or 5 μg/mL CIP. Selected samples were treated with 1 mM ADO. Data are the mean of three biological replicates ± SEM. *, *P < *0.05; **, *P < *0.01; ***, *P < *0.001, as assessed by Student's *t* test. ADO, adenosine; ND, no CFU detected; NS, not statistically significant; VEH, vehicle control.

To further investigate the role of the ETC in the ADO-dependent increased antibiotic killing phenotype, we examined an Δ*atpB S.* Typhimurium mutant that lacks a critical subunit of the ATP synthase (43) and a small colony variant that bears mutations in the *nuo* operon (Δ*nuo::km*) and *ndh* gene (Δ*ndh::FRT*) encoding NDH-I and NDH-II NADH dehydrogenases, respectively (44). Addition of ADO significantly (*P < *0.01) increased GEN killing of the Δ*atpB* mutant, though the effect was less than in the wild-type strain. In contrast, ADO did not increase GEN killing of the Δ*nuo* Δ*ndh* mutant (Fig. 5A). Moreover, whereas ADO enhanced lethality of CIP in stationary-phase Salmonella, ADO did not significantly (*P < *0.05) potentiate CIP killing of Δ*atpB or* Δ*nuo* Δ*ndh* mutants (Fig. 5B). These results highlight the importance of an intact ETC for the full impact of ADO on antibiotic killing. The inability of ADO to enhance antibiotic killing of Salmonella deficient in NDH-I and NDH-II NADH dehydrogenases suggests that electron flux through this complex of the ETC is critical for the potentiation of antibiotic killing by ADO. On the other hand, ADO-driven enhanced killing of the Δ*atpB* mutant suggests a mechanism independent of oxidative phosphorylation.

### ADO alters nucleotide metabolism and antibiotic killing in S. aureus.

**FIG 6:**
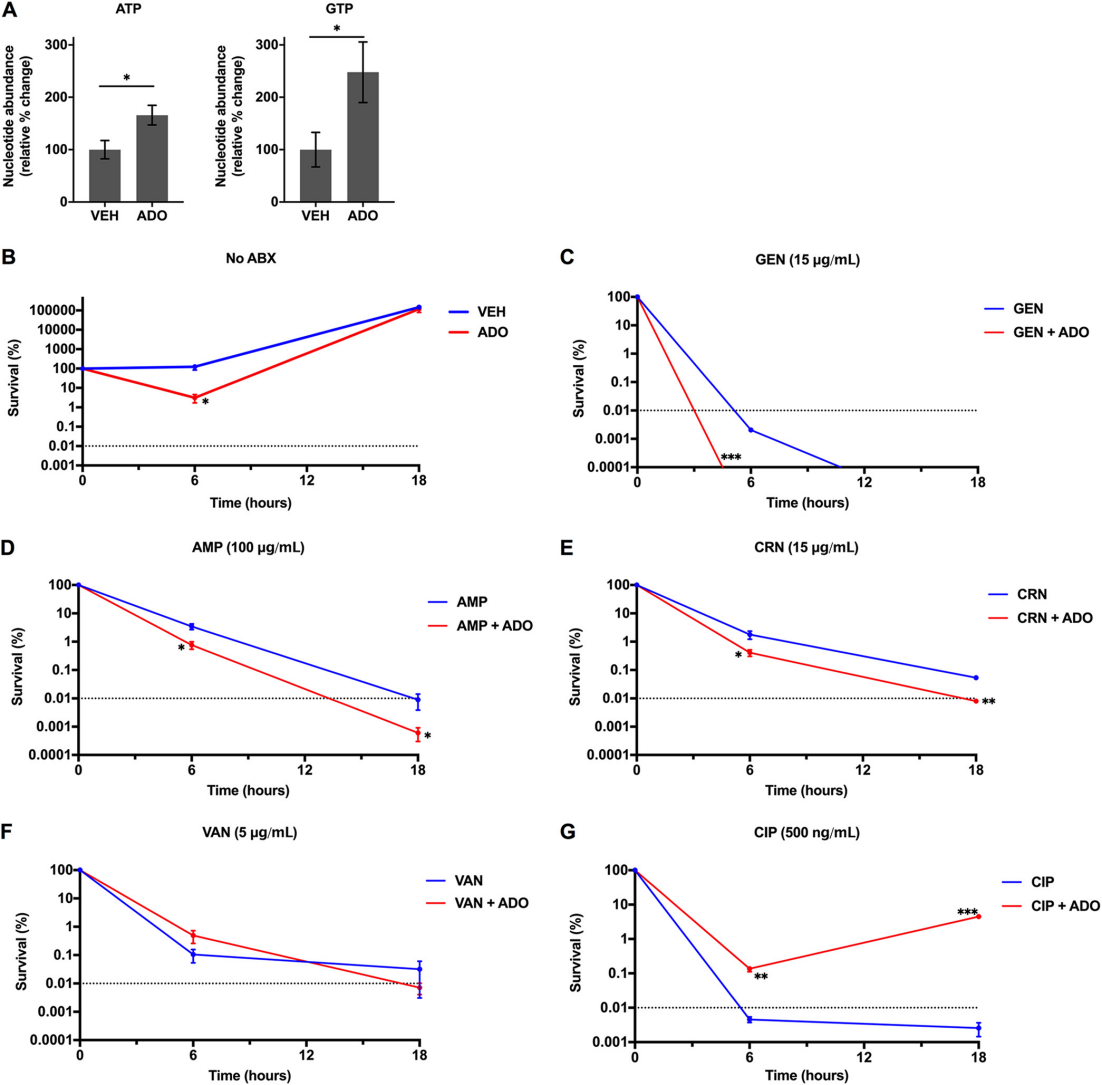
ADO sensitizes S. aureus to antibiotic killing. (A) S. aureus intracellular nucleotide concentrations 15 min after downshift from LB broth into M9 minimal medium. Selected samples were treated with vehicle or 1 mM ADO. (B to G) To establish MDK values, antibiotic killing was determined in S. aureus cultures after downshifting from exponential-phase LB broth to M9 medium supplemented with 0.2% Casamino Acids, 5 μg/mL thiamine, and 2 μg/mL nicotinic acid, without glucose. Selected samples were treated with vehicle or 1 mM ADO in the presence of 15 μg/mL GEN, 100 μg/mL AMP, 15 μg/mL CRN, 5 μg/mL VAN, 500 ng/mL CIP, or antibiotic vehicle control. Data are the mean of three biological replicates ± SEM. *, *P < *0.05; **, *P < *0.01; ***, *P < *0.001, as assessed by Student's *t* test. Dotted line indicates MDK_99.99_ threshold. Asterisk denotes significant difference between treatment groups. ABX, antibiotics; ADO, adenosine; AMP, ampicillin; CIP, ciprofloxacin; CRN, ceftriaxone; GEN, gentamicin; VAN, vancomycin; VEH, vehicle control.

We then expanded our investigations to the Gram-positive bacterium Staphylococcus aureus. Amino acid deprivation of S. aureus results in a rapid consumption of the substrate GTP and a concomitant accumulation of (p)ppGpp by RSH (RelA/SpoT homolog) (45). To examine ATP and GTP in S. aureus following ADO treatment, S. aureus cultures were subjected to a nutrient downshift into glucose-free M9 minimal media supplemented with ADO for 15 min. Treatment of S. aureus with ADO significantly increased ATP and GTP levels (Fig. 6A). Conlon et al. demonstrated that decreased ATP levels in S. aureus correlates with increased persister formation (46).

We tested antibiotic lethality against S. aureus using several classes of antibiotics. For these experiments, S. aureus was shifted from exponential-phase growth into M9 minimal media supplemented with nicotinic acid, thiamine, and Casamino Acids. Unlike E. coli and *S.* Typhimurium, this medium composition allowed the bacteria to grow over 18 h. Even in the absence of antibiotics, inclusion of 1 mM ADO resulted in decreased CFU at 6 h, but the numbers of S. aureus increased by 18 h (Fig. 6B). The addition of ADO together with either GEN, ampicillin, or ceftriaxone increased killing activity compared to control groups treated with antibiotics only. Accordingly, ADO shortened the MDK_99.99_ for these antibiotic classes (Fig. 6C to E). We did not observe significant changes for vancomycin-treated groups (Fig. 6F). Unexpectedly, the combination of CIP and ADO provided a protective effect at both time points compared to the CIP-only group (Fig. 6G). Overall, these data indicate that ADO can enhance antibiotic lethality against S. aureus for multiple classes of antibiotics. However, ADO can also play a protective role in the case of a fluoroquinolone. Under a more complex medium, the opposing effects of ADO on survival depend on the antibiotic class. Identifying and understanding antibiotic class-specific mechanisms may provide insights for developing improved therapies. While the magnitude of impact ADO has on antibiotic lethality in S. aureus is not as large as the impact seen in E. coli or *S.* Typhimurium, it does demonstrate that ADO can enhance antibiotic lethality in an important Gram-positive human pathogen.

## DISCUSSION

Our investigations demonstrate that ADO significantly alters nucleotide metabolism in E. coli to suppress the stringent response, increase PMF, promote cellular respiration, and drive cellular energetics in starving bacteria. An important consequence of this ADO-induced metabolic shift is a dramatic increase in antibiotic lethality against starvation-triggered tolerant and stationary-phase persister bacteria in the absence of significant growth. These effects suggest that during starvation, ADO induces a state that has been called “persister awakening” (10).

In the present work, we focused on the ADO-driven shifts in nucleotide metabolism, the consequences of which drive potentiation of GEN, CIP, ampicillin, and ceftriaxone killing of several Gram-positive and Gram-negative human pathogens. (p)ppGpp deficiency, defective purine salvage, loss of complex I, or ATP synthase partially protects bacteria from ADO-driven potentiation of antibiotic killing, suggesting that exogenous ADO influences two pathways of persister formation, the stringent response and energy-producing pathways. Disruption of the stringent response dysregulates cell metabolism, while concurrent increases in PMF and respiration by ADO play a more profound role in potentiating antibiotic lethality. Recent work highlighted the importance of purine nucleotide pools and *de novo* purine biosynthesis in antibiotic lethality (39). We observe that metabolically engaging purine salvage potentiates the toxicity of different classes of antibiotics against diverse species of bacteria. Our research suggests that the effects that ADO exerts on cellular processes and the corresponding resensitization to antibiotics may be evolutionarily conserved.

Allison et al. have shown that metabolites such as glucose, mannitol, fructose, and pyruvate can enhance GEN-mediated killing of E. coli in minimal media by fueling central bacterial metabolism (7). They found that these metabolites drove PMF and respiration to potentiate killing by GEN specifically. There are important differences to consider between their work and ours. First, we observe that ADO enhances killing by GEN and CIP, whereas the carbon sources used by Allison et al. specifically potentiate GEN killing. Similar to the metabolites reported by Allison et al., we do not observe potentiation of antibiotic killing by ADO in the presence of CCCP, suggesting that it is dependent on PMF. Our data support the conclusion that the influence of ADO on antibiotic killing is ETC dependent and can occur in aerobic and anaerobic conditions. However, unlike the previously reported metabolites, potentiation of antibiotic killing by ADO is not dependent on the presence of a terminal electron acceptor such as O_2_ or nitrate. Thus, our work supports a model wherein ADO metabolism not only activates central metabolism but also stimulates ETC activity. The specific mechanism by which activation of ADO salvage potentiates GEN and CIP and the electron donors and acceptors generated through ADO metabolism are under investigation.

A novel finding in the current study is that ADO enhances antibiotic killing without stimulating bacterial growth. Compelling arguments have been made that the reduction in growth rate alone accounts for antibiotic tolerance in persistent bacteria and that other metabolic shifts, including activation of (p)ppGpp-mediated signaling and depletion of ATP, are not sufficient to maintain tolerance if the rate of growth is increased (14, 38). It is clear from these and other studies that an increase in growth rate correlates with resensitization to antibiotics. In contrast, the reversion of antibiotic tolerance by ADO is not the result of stimulation of growth. In this context, our findings are even more remarkable considering the observation that ADO potentiates antibiotic lethality even though it suppresses growth of Salmonella and E. coli (16) (Fig. 1A). Under the conditions of the antibiotic survival assays presented here, we saw no significant increase in growth in the presence ADO with or without antibiotics, even though ADO reduced survival in antibiotics by up to 5 orders of magnitude. Though the literature on ADO as a sole carbon and nitrogen source for E. coli is limited, our data show that the growth rate in our conditions is below our limits of detection and is incongruent with the magnitude of bacterial killing reported herein. Intuitively, increased growth rate generally requires increased cellular metabolism. On the other hand, increased metabolism does not necessarily lead to cellular replication. In fact, others have shown that metabolism and growth rate can be uncoupled (8, 10, 14, 15, 47). Our evidence shows that ADO activates cellular metabolism while not permitting or, in some instances, slowing growth. This leads to the conclusion that growth is not the dominant mechanism underlying potentiation of antibiotics by ADO over the time period examined. More broadly, our data provide evidence that activation of cellular metabolism without stimulation of growth can be sufficient to reduce antibiotic tolerance.

We anticipate that each antibiotic will be potentiated through class-specific mechanisms, some of which have been previously described. For example, ADO-driven increases in PMF drive GEN uptake, effectively delivering more drug to its ribosomal target (9, 48). CIP-induced double-strand DNA breaks are known to activate the SOS response, an adaptive program associated with antibiotic tolerance (49). While there is evidence that the stringent response regulates expression of the SOS response (50), further investigation is necessary to determine whether ADO could inhibit the latter stress program independently of its actions on the stringent response to abrogate CIP tolerance. The global enhancement of cellular energetics following ADO may allow for awakening of persisters, thus improving the bactericidal activity of various classes of antibiotics against clinically diverse bacteria. It is noteworthy that mucosal surfaces generate large amounts of extracellular ADO (16). Host-derived ADO may sensitize bacteria to antibiotic killing. However, ADO itself is likely not a feasible antimicrobial adjuvant due to its rapid uptake by host tissues and potentially detrimental cardiovascular side effects (51). Rather, approaches to increase host extracellular ADO or alter bacterial nucleotide salvage and metabolism to promote cellular respiration and awaken persister populations may provide opportunities to decrease the formation of antimicrobial resistance.

## MATERIALS AND METHODS

### Microbial strains.

E. coli K-12 BW25113 wild type and E. coli K-12 BW25113 Δ*deoD* were obtained from the commercially available Keio collection (Horizon Discovery). E. coli K-12 MG1655 Δ*relA* Δ*spoT* and its isogenic control have previously been described (52). *S.* Typhimurium ATCC 14028s and Staphylococcus aureus subsp. *aureus* ATCC 12600 were obtained from ATCC. *S.* Typhimurium mutants were obtained from the selected publications (43, 44). Strains are listed in Table S2B in the supplemental material; plasmids are listed in Table S2C. Unless otherwise specified, for each experiment, strains were cultured and maintained in LB broth and LB agar under aerobic conditions at 37°C and 250 rpm agitation for liquid cultures. Appropriate antibiotic selection was used for each strain in both LB broth and LB agar. Stock strains were stored at −80°C with 15% glycerol. All experiments were performed with bacteria subcultured 1:50 from 3 mL overnight cultures prepared in 14 mL round-bottom snap-cap culture tubes, unless otherwise specified. Bacteria were washed and pelleted at 4000g for 5 min in 1.5-mL Eppendorf tubes. For all experiments, bacteria were diluted in respective media using OD_600_ to determine culture density.

### Defined minimal media.

M9 medium (48 mM Na_2_HPO_4_ · 7H_2_O, 22 mM KH_2_PO_4_, 8.56 mM NaCl, 18.69 mM NH_4_Cl, 1 mM MgSO_4_, 100 μM CaCl_2_, and 50 μM FeSO_4_ · 7H_2_O, pH 7.1) was also purchased through Teknova (catalog no. M8005). M9 used with S. aureus is the M9 listed above with 0.2% Casamino Acids, 2 μg/mL nicotinic acid, and 5 μg/mL thiamine MOPS [40 mM MOPS buffer, 4 mM tricine, 0.4% d-glucose, 40 μg/mL of each amino acid except serine, 2 mM K_2_HPO_4_, 10 μM FeSO_4_ · 7H_2_O, 9.5 mM NH_4_Cl, 276 μM K_2_SO_4_, 500 nM CaCl_2_, 50 mM NaCl, 525 μM MgCl_2_, 2.9 nM (NH_4_)_6_Mo_7_O_24_ · 4H_2_O, 400 nM H_3_BO_3_, 30 nM CoCl_2_, 9.6 nM CuSO_4_, 80.8 nM MnCl_2_, and 9.74 nM ZnSO_4_, pH 7.2]. PBS, pH 7.4, was purchased from Gibco (catalog no. 10010023). PBS was used in many experiments to eliminate confounding growth influence of ADO in M9 minimal medium.

### Growth curve assay.

Bacteria were subcultured 1:50 from an overnight stock. Subcultures were grown for ~3.5 hours until late exponential phase, as described above. At an optical density at 600 nm (OD_600_) of ~0.4, 1 mL of cells were isolated in a 1.5-mL Eppendorf tube and washed three times with M9 minimal medium; centrifugation was for 5 min at 4,000 × *g*. Bacteria were then diluted in M9 medium to an OD_600_ of 0.001, and 135 μL of culture was applied to a 96-well ultralow binding plate with 15 μL of vehicle or treatment. OD_600_ was taken every 10 min, and plates were maintained at 37°C with 768 rpm agitation.

### ^32^P TLC purine nucleotide assay.

Bacteria grown overnight in LB broth were washed 3 times in MOPS minimal medium containing 40 μg/mL of all amino acids and subcultured 1:100 in MOPS medium containing all amino acids at 40 μg/mL; cells were then labeled with ^32^P orthophosphate at 10 μCi/mL overnight. The next day, bacteria were washed 3 times in M9 medium and resuspended into respective conditions, which are defined in figure legends. Nucleotides were then extracted using ice-cold 3.5-M formic acid; 500 μL culture was added to 200 μL formic acid for a final concentration of 1 M formic acid. Extraction was performed on ice for 20 min. Cell debris was pelleted in a tabletop centrifuge at 13,000 rpm for 5 min. We applied 2-μL spots 4 times to a polyethyleneimine (PEI)-cellulose plate, with 8 μL total volume per spot. (p)ppGpp-null bacteria and nutrient-rich conditions were used as negative controls; amino acid starvation conditions were used as positive controls. Plates were resolved in 1.5 M KH_2_PO_4_, pH 3.4, for ~80 min. Plates were exposed to a phosphorimage screen for 24 h and subsequently analyzed on a Bio-Rad phosphorimager with 50 μm resolution.

### GFP promoter assay.

Bacteria were subcultured 1:50 from an overnight stock. Subcultures were grown for ~2 to 3 h until exponential phase. Bacteria were then washed three times in PBS and then resuspended into PBS until an OD_600_ of 0.2 was reached. We added 150 μL of culture to each well of a 96-well black wall clear-bottom plate containing respective treatment conditions. Green fluorescent protein (GFP) signal and OD_600_ were analyzed every 5 min using a BioTek Instruments Synergy H1 microplate reader at 37°C with agitation.

### Respiration assay.

Bacteria were subcultured 1:50 from an overnight stock. Subcultures were grown for ~2 to 3 h until exponential phase. One milliliter of bacteria was then washed three times in PBS and then resuspended into PBS to an OD_600_ of 0.2. One milliliter of culture was added to each well of a 12-well OxoDish plate containing respective treatment conditions. Plates were analyzed every minute using PreSens OxoDish at 37°C with agitation.

### XTT assay.

Bacteria were subcultured 1:50 from an overnight stock. Subcultures were grown for ~2 to 3 h until exponential phase. One milliliter of bacteria was then washed three times in PBS and then resuspended into PBS to an OD_600_ of 0.05. For anaerobic cultures, an overnight stationary culture was washed three times in PBS prior to treatment. Cells were suspended in PBS containing 200 μg/mL XTT {2,3-bis(2-methoxy-4-nitro-5-sulfophenyl)-5-((phenylamino)carbonyl)-2H-tetrazolium hydroxide} with 5.5 μg/mL menadione. We added 100 μL of culture to a 96-well plate containing respective treatment conditions. Plates were analyzed with OD_460_ to OD_660_ to measure XTT product accumulation. Anaerobic samples were brought to the plate reader in an airtight anaerobic case. For kinetic assays, plates were maintained at 37°C with 768 rpm agitation and read every 9 min.

### HPLC and LC-MS assays.

Bacteria were subcultured 1:50 from an overnight stock. Subcultures were grown for ~2 to 3 h until exponential phase. One milliliter of bacteria was then washed three times in PBS and then resuspended into PBS to an OD_600_ of 0.4. For anaerobic cultures, an overnight stationary culture was washed three times in PBS prior to treatment, and samples were snap frozen in liquid nitrogen. Samples were then extracted using ice-cold 3.5 M formic acid, and 500 μL culture was added to 200 μL formic acid for a final concentration of 1 M formic acid. Extraction was performed on ice for 20 min. Samples were frozen to −80°C and then thawed and dried at room temperature using a SpeedVac centrifuge. Samples were then suspended in 150 μL mobile-phase buffer (75 mM monobasic potassium phosphate, 10 mM tetrabutylammonium hydrogen sulfate, pH 6.25, with triethylamine). Samples were then ultrafiltered using Sartorius Vivaspin 500 5,000-Da molecular weight cutoff (MWCO) polyethersulfone (PES) filters. We injected 75 μL of sample, and metabolites were quantitated by high-performance liquid chromatography (HPLC) as previously described (53) with ATP and GTP absorbance spectra and retention times verified by coinjection with authentic standards. The metabolites were quantitated from calibration curves ranging from 100 nM to 5 mM. LC-MS samples were quantitated as previously described (54). Metabolites were detected by the masses of their negatively charged ions (ATP, 346 *m/z*; succinate, 117 *m/z*). Retention times and *m/z* were verified by coinjection with authentic standards. Analysis of samples and curves was performed blind. An Agilent Technologies 1260 Infinity system was used for HPLC analysis. LC-MS was performed on an Agilent Technologies 1260 Infinity II LC/MSD iQ with electrospray ionization (ESI) mass detection.

### CFU antibiotic killing assay.

Bacteria were subcultured 1:50 from an overnight stock. Subcultures were grown for ~2 to 3 h until the exponential phase. One milliliter of bacteria was then washed three times with PBS and then resuspended into PBS or M9 medium to an OD_600_ of 0.05. We added 3 mL of culture to 14-mL round-bottom culture tubes, and respective treatments were applied. For the persister assay, cells were grown to stationary phase overnight in LB broth and used the following morning. All treatment groups for a given strain were established from the same inoculum to minimize starting cell density variability between treatment groups. For all experiments, individual replicate experiments were normalized to starting inoculum to account for variability between experiments. Bacteria were cultured for the desired amount of time at 37°C with 250 rpm agitation. Culturing in minimal versus nutrient-rich media influenced the MIC for a bacterial strain. Based on this, we did not use fold increase over MIC as determined by standard microplate dilution in nutrient-rich LB broth for every experiment (Table S2A). For some experiments, the antibiotic concentrations used were determined empirically based on specific experimental conditions and durations. Following treatment, 500 μL of bacterial culture was added to 1.5-mL Eppendorf tubes containing 500 μL of PBS. Cells were washed three or four times (depending on antibiotic concentration) with PBS to remove antibiotics and then suspended in PBS to the initial culture volume of 500 μL. For zero-hour CFU, bacteria were serial diluted in PBS at 1:5, 10-μL spots were applied to LB agar to enumerate CFU. For antibiotic killing conditions, bacteria were serial diluted in PBS 1:3, and 10-μL spots were applied to LB agar to enumerate CFU. Plates were incubated overnight at 37°C. CFU were counted manually the following day and held for at least 2 more days to allow for possible emergence of slow-growing persister bacteria, which were added to initial counts. Survival assays using 50 μM carbonyl cyanide *m*-chlorophenyl hydrazone (CCCP) (Sigma; catalog no. C2759) were performed aerobically as described above. CCCP was administered simultaneously with the ADO and antibiotic (ABX) treatments. For anaerobic survival assays, bacteria were introduced into the anaerobic chamber 1 week prior to the experiment and serial passaged in LB broth. Anaerobic survival was conducted in the anaerobic chamber with the same approach as described above. All reagents were stored under anaerobic conditions to eliminate the introduction of O_2_. Colorimetric O_2_ indicators were kept in the anaerobic chamber during experiments to confirm anaerobic conditions.

### Statistical analysis.

Statistical tests and sample sizes are indicated within the figure legends. A minimum of three biological experimental repeats were conducted for all experiments. Blinding was used to minimize bias during HPLC and LC-MS analysis. No data points were excluded from analysis. All statistical tests were performed with a two-tailed analysis unless otherwise indicated. Significance threshold for α was set at 0.05. Error bars indicate standard error of the mean unless otherwise indicated. Microsoft Excel was used to organize raw data sets. GraphPad Prism 8 was used for statistical analysis and figure generation. Bio-Rad Image Lab was used for phosphorimage analysis.
