# Supplementary figures and images for: Adenosine Awakens Metabolism to Enhance Growth-Independent Killing of Tolerant and Persister Bacteria across Multiple Classes of Antibiotics
Source: mBio. 2022 May 16;13(3):e00480-22. doi: 10.1128/mbio.00480-22 (PMC9239199; doi:10.1128/mbio.00480-22)

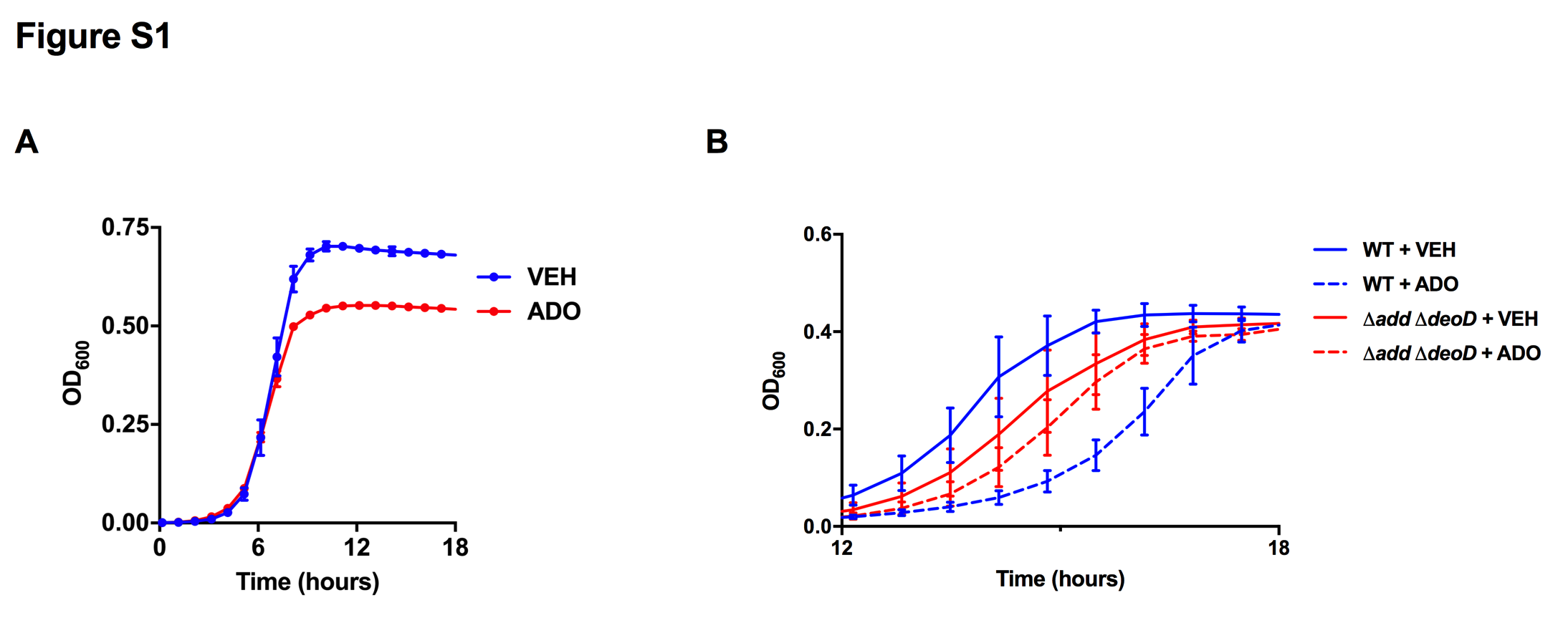

Supplement: FIG S1 [file mbio.00480-22-s0001.tif]

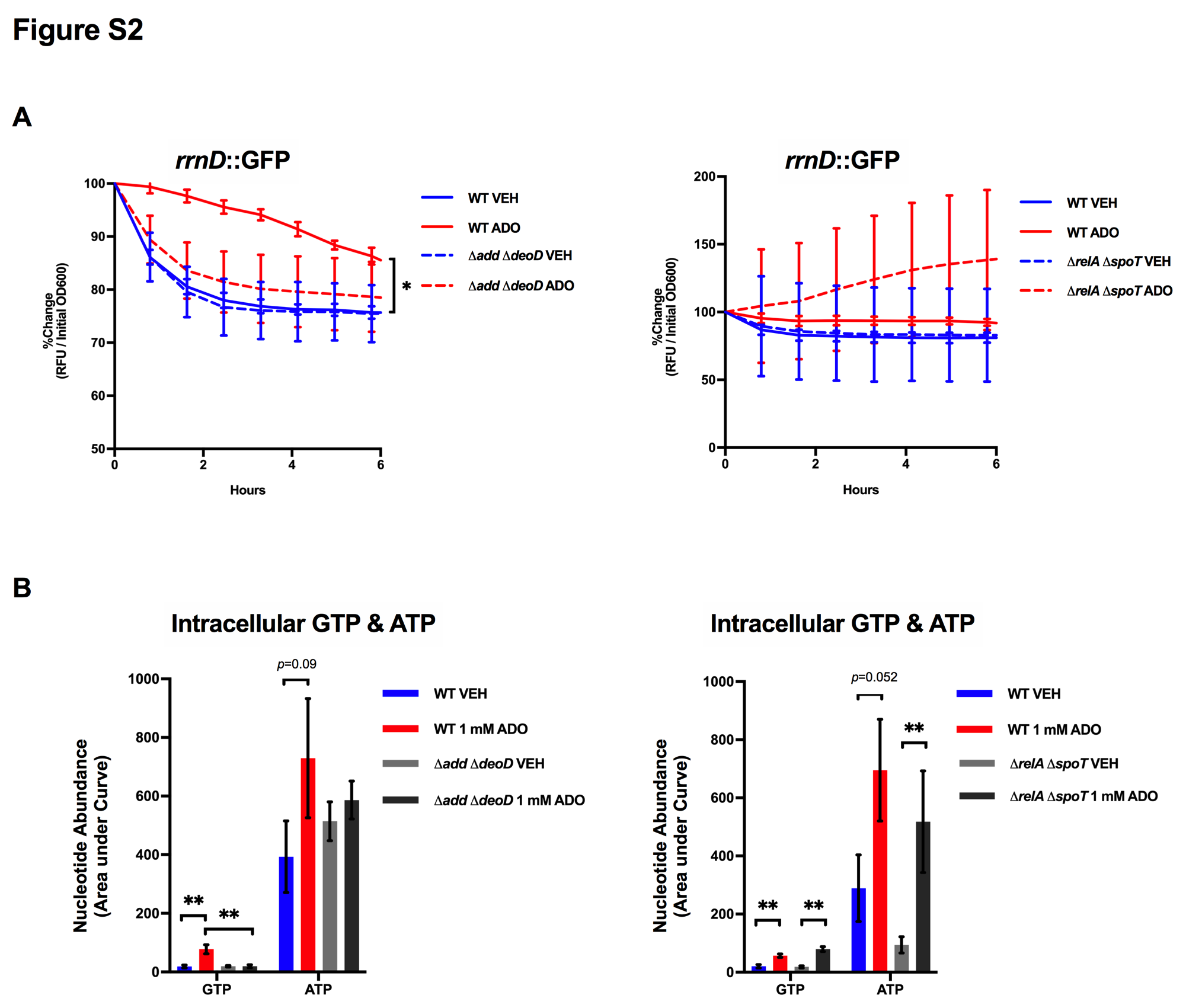

Supplement: FIG S2 [file mbio.00480-22-s0002.tif]

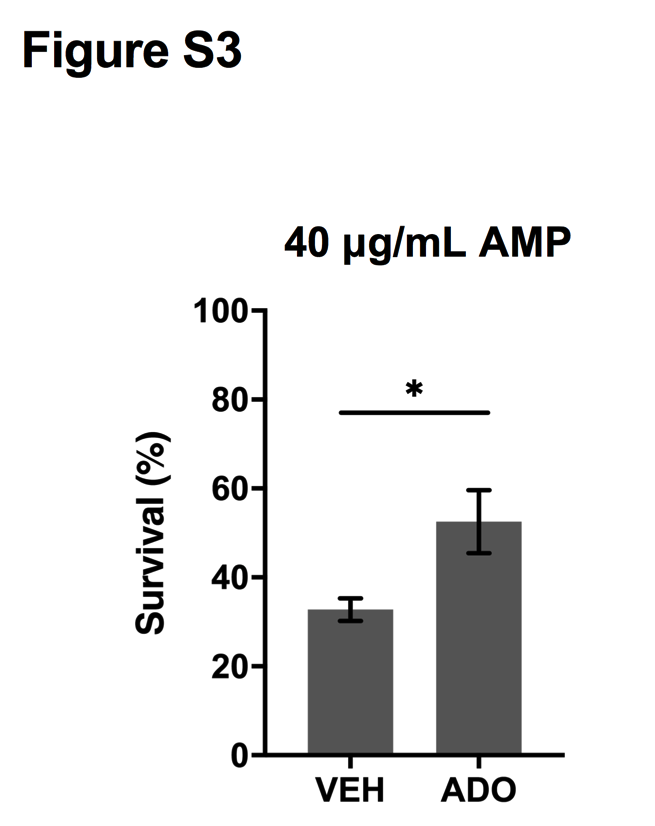

Supplement: FIG S3 [file mbio.00480-22-s0003.tif]

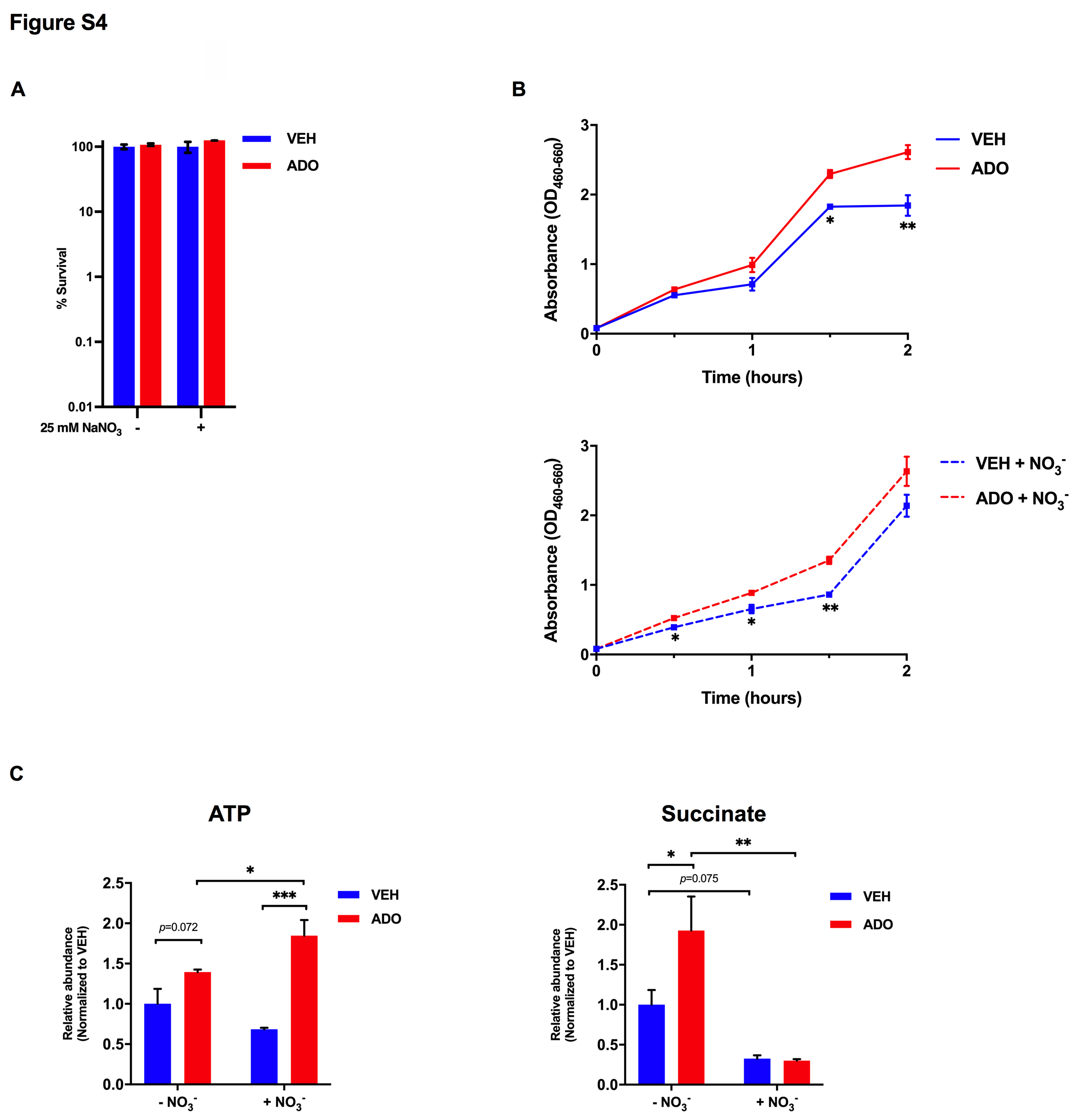

Supplement: FIG S4 [file mbio.00480-22-s0004.tif]

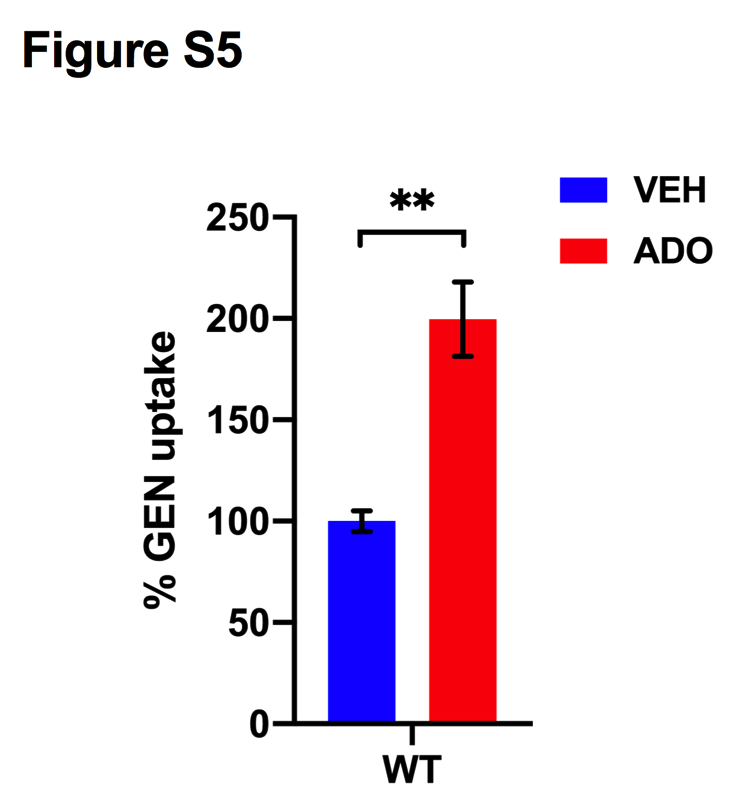

Supplement: FIG S5 [file mbio.00480-22-s0005.tif]

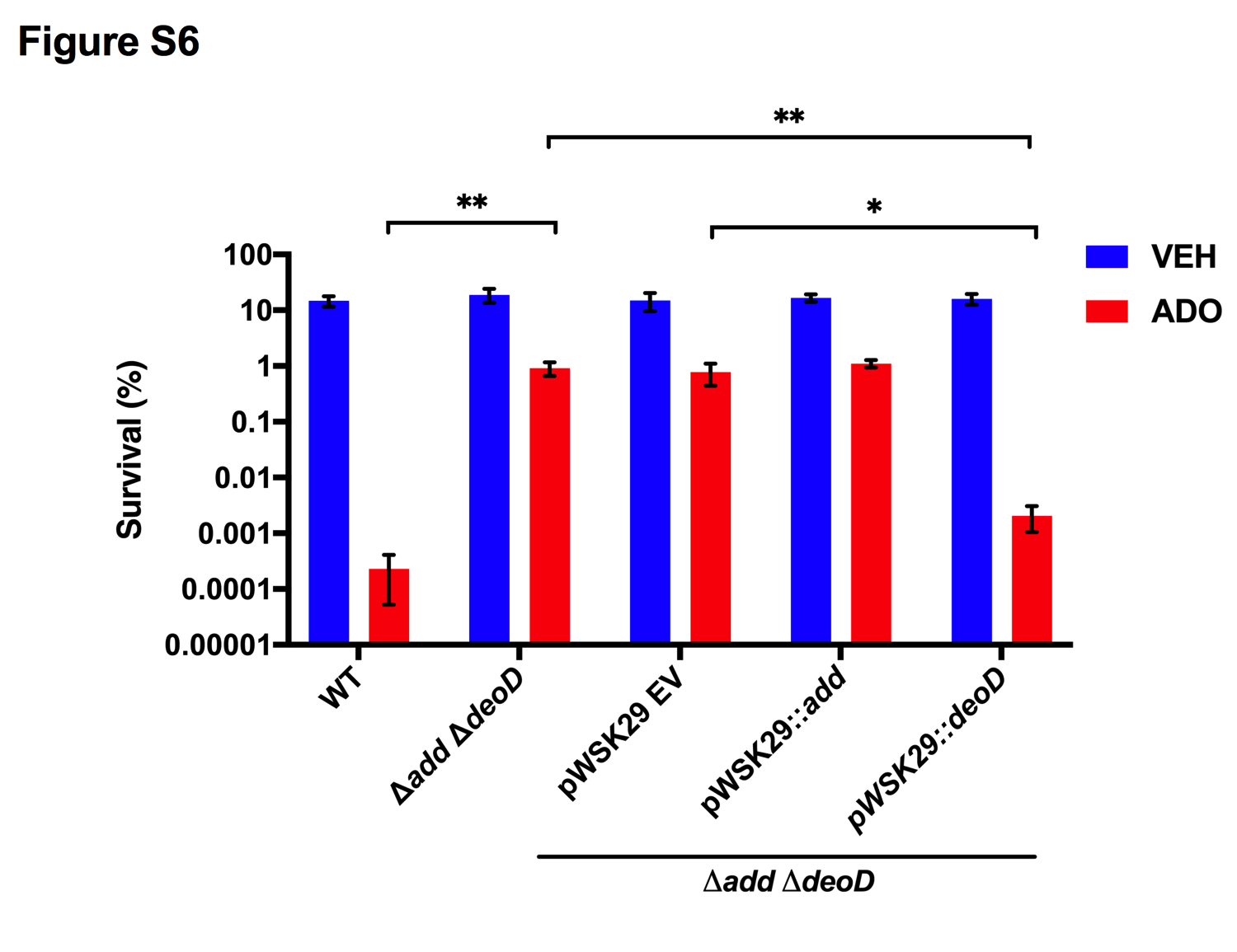

Supplement: FIG S6 [file mbio.00480-22-s0006.tif]

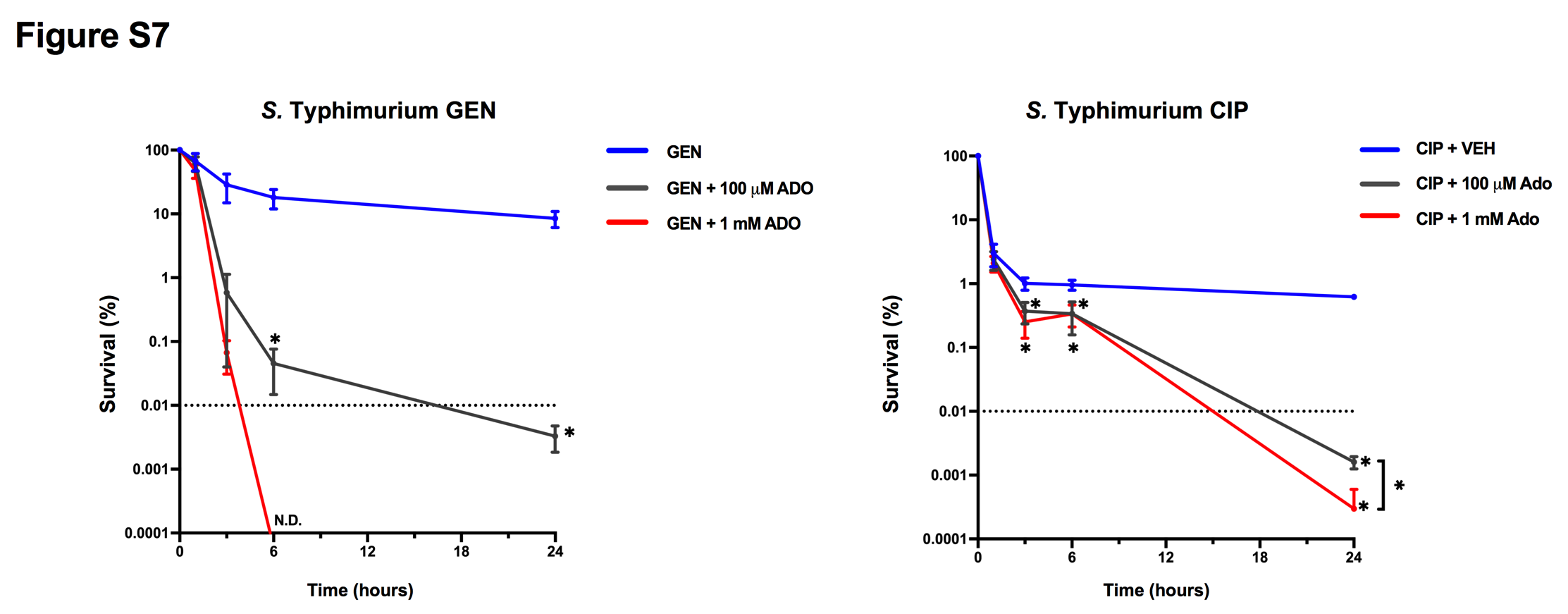

Supplement: FIG S7 [file mbio.00480-22-s0007.tif]
