# Supplementary material for: Adenosine Awakens Metabolism to Enhance Growth-Independent Killing of Tolerant and Persister Bacteria across Multiple Classes of Antibiotics
Source: mBio. 2022 May 16;13(3):e00480-22. doi: 10.1128/mbio.00480-22 (PMC9239199; doi:10.1128/mbio.00480-22)
Supplement: TABLE S1 [file mbio.00480-22-s0008.pdf]

Supplemental Table 1: Intracellular amino acid concentrations (nmol/billion cells)

	NT			Ado			Median		Ado / NT	
**Compound**	**1**	**2**	**3**	**1**	**2**	**3**	**NT**	**Ado**	**Fold change**	**p-value**
Alanine	3.51	4.00	3.66	3.86	3.57	3.94	3.66	3.86	1.06	0.731
Arginine	0.66	0.63	0.67	0.46	0.54	0.53	0.66	0.53	0.80	0.005
Aspartate	1.22	0.53	0.65	0.64	0.52	0.57	0.65	0.57	0.88	0.354
Glutamate	28.93	32.28	29.97	28.61	25.48	25.17	29.97	25.48	0.85	0.055
Histidine	0.12	0.12	0.11	0.07	0.07	0.15	0.12	0.07	0.61	0.510
Isoleucine	0.17	0.17	0.17	0.15	0.13	0.16	0.17	0.15	0.88	0.050
Leucine	0.14	0.11	0.11	0.09	0.09	0.13	0.11	0.09	0.83	0.444
Lysine	3.16	2.85	3.37	2.98	2.52	2.57	3.16	2.57	0.81	0.105
Methionine	0.01	0.01	0.01	0.01	0.01	0.01	0.01	0.01	0.84	0.379
Phenylalanine	0.41	0.40	0.41	0.36	0.33	0.38	0.41	0.36	0.89	0.022
Proline	0.51	0.54	0.55	0.53	0.52	0.57	0.54	0.53	0.98	0.717
Serine	0.15	0.11	0.19	0.22	0.08	0.50	0.15	0.22	1.50	0.399
Threonine	0.10	0.11	0.11	0.11	0.10	0.18	0.11	0.11	1.00	0.399
Tyrosine	0.32	0.32	0.33	0.34	0.32	0.35	0.32	0.34	1.05	0.239
Valine	0.49	0.41	0.41	0.48	0.39	1.22	0.41	0.48	1.16	0.380
