# Supplementary material for: Adenosine Awakens Metabolism to Enhance Growth-Independent Killing of Tolerant and Persister Bacteria across Multiple Classes of Antibiotics
Source: mBio. 2022 May 16;13(3):e00480-22. doi: 10.1128/mbio.00480-22 (PMC9239199; doi:10.1128/mbio.00480-22)
Supplement: TABLE S2 [file mbio.00480-22-s0009.pdf]

Supplemental Table 2: Strain information

S2A: Strain MIC

Strain	Gentamicin	Ciprofloxacin	Ampicillin	Ceftriaxone	Vancomycin
E. Coli K-12 BW25113	4	0.03125 ng/mL	4		
S. Typhimurium 14028s	4	0.03125 ng/mL	4		
S. aureus 12600	16	250 ng/mL	1	500 ng/mL	1

S2B: Strains

Strain	Relevant genetic marker(s) or features	Source, reference or construction
E. coli strains		
K-12 BW25113	F-, Δ(araD-araB)567, ΔlacZ4787 (::rrnB-3), λ-, ΔguaB757::kan , rph-1 , Δ(rhaDrhaB) 568 , hsdR514	Keio collection
K-12 BW25113		Transformation, select $strep^R$
K-12 BW25113 AD	BW25113 *Δadd ΔdeoD*	Transformation, select $strep^R$
WT rrnB:GFP	BW25113 *rrnB* P1*:gfp*	Transformation, select $strep^R$, select Kan^R
AD rrnB:GFP	BW25113 *Δadd ΔdeoD rrnB* P1*:gfp*	Transformation select $strep^R$, select Kan^R
WT rrnD:GFP	BW25113 *rrnD* P1*:gfp*	Transformation select $strep^R$, select Kan^R
AD rrnD:GFP	BW25113 *Δadd ΔdeoD rrnD* P1*:gfp*	Transformation select $strep^R$, select Kan^R
AD pWSK29 EV	BW25113 *Δadd ΔdeoD,* pWSK29	Transformation select Amp^R
AD pWSK29::*add*	BW25113 *Δadd ΔdeoD,* pWSK29::*add*	Transformation select Amp^R
AD pWSK29::*deoD*	BW25113 *Δadd ΔdeoD,* pWSK29::*deoD*	Transformation select Amp^R
CF1648	F-, λ-, rph-1	Cashel lab MG1655 WT
CF1693	*CF1648 ΔrelA::kan ΔspoT::cam*	Cashel lab (52)
AVT K12 WT rrnB:GFP	CF1648 *rrnB* P1*:gfp*	Transformation, select Kan^R
AVT K12 RS rrnB:GFP	CF1693 *ΔrelA ΔspoT rrnB* P1*:gfp*	Transformation, select Kan^R
AVT K12 WT rrnD:GFP	CF1648 *rrnD* P1*:gfp*	Transformation, select Kan^R
AVT K12 RS rrnD:GFP	CF1693 *ΔrelA ΔspoT rrnD* P1*:gfp*	Transformation, select Kan^R
Salmonella strains		
14028s	*S.* Typhimurium	ATCC
14028s AV17122	*ΔatpB*	(43)
14028s AV0438	*Δnuo Δndh*	(44)
S. aureus strain		
ATCC 12600	NCTC 8532	ATCC

S2C: Plasmids

Plasmid	Description	Reference
pE-FLP	ori *repA101, flp,* Amp^R	Addgene plasmid #45978
pMSs201 rrnBP1-GFP	*rrnBP1:gfp,* Kan^R	Dharmacon Horizon Discovery
pMSs201 rrnDP1-GFP	*rrnDP1:gfp,* Kan^R	Dharmacon Horizon Discovery
pORTMAGE	pBBR1 oriV, *mutL E32K*, λ rep (ts), *beta, exo*, Cm^R	Addgene plasmid #72679
pWSK29	Amp^R	Addgene plasmid #172972
pWSK29::*add*	*add,* Amp^R	
pWSK29::*deoD*	*deoD,* Amp^R	
